# Discovery and Anti-Inflammatory Activity of a Cyanobacterial Fatty Acid Targeting the Keap1/Nrf2 Pathway

**DOI:** 10.3390/md21110553

**Published:** 2023-10-25

**Authors:** Fatma H. Al-Awadhi, Emily F. Simon, Na Liu, Ranjala Ratnayake, Valerie J. Paul, Hendrik Luesch

**Affiliations:** 1 Department of Medicinal Chemistry and Center for Natural Products, Drug Discovery and Development (CNPD3), University of Florida, Gainesville, FL 32610, USA or fatma.h@ku.edu.kw (F.H.A.-A.); simonemily@ufl.edu (E.F.S.); mls_liun@ujn.edu.cn (N.L.); rratnayake@cop.ufl.edu (R.R.); 2 Department of Pharmaceutical Chemistry, Faculty of Pharmacy, Kuwait University, P.O. Box 24923, Safat 13110, Kuwait; 3 School of Biological Science and Technology, University of Jinan, Jinan 250022, China; 4 Smithsonian Marine Station, Fort Pierce, FL 34949, USA; paul@si.edu

**Keywords:** marine cyanobacteria, fatty acids, inflammation, RNA sequencing, NF-κB, immune function, multiple sclerosis

## Abstract

The monounsaturated fatty acid 7(*E*)-9-keto-hexadec-7-enoic acid (**1**) and three structurally related analogues with different oxidation states and degrees of unsaturation (**2**–**4**) were discovered from a marine benthic cyanobacterial mat collected from Delta Shoal, Florida Keys. Their structures were elucidated using NMR spectroscopy and mass spectrometry. The structure of **1** contained an α,*β*-unsaturated carbonyl system, a key motif required for the activation of the Keap1/Nrf2−ARE pathway that is involved in the activation of antioxidant and phase II detoxification enzymes. Compounds **1**–**4** were screened in ARE-luciferase reporter gene assay using stably transfected HEK293 cells, and only **1** significantly induced Nrf2 activity at 32 and 10 µM, whereas **2**–**4** were inactive. As there is crosstalk between inflammation and oxidative stress, subsequent biological studies were focused on **1** to investigate its anti-inflammatory potential. Compound **1** induced *Nqo1*, a well-known target gene of Nrf2, and suppressed *iNos* transcript levels, which translated into reduced levels of nitric oxide in LPS-activated mouse macrophage RAW264.7 cells, a more relevant model for inflammation. RNA sequencing was performed to capture the effects of **1** on a global level and identified additional canonical pathways and upstream regulators involved in inflammation and immune response, particularly those related to multiple sclerosis. A targeted survey of marine cyanobacterial samples from other geographic locations, including Guam, suggested the widespread occurrence of **1**. Furthermore, the previous isolation of **1** from marine diatoms and green algae implied a potentially important ecological role across marine algal eukaryotes and prokaryotes. The previous isolation from sea lettuce raises the possibility of dietary intervention to attenuate inflammation and related disease progression.

## 1. Introduction 

Inflammation is a process that involves the activation of immune and non-immune cells as a protective mechanism in response to exposure to toxins, pathogens and infection, as well as tissue injury [1]. Although intermittent or acute inflammation is crucial for tissue repair, recovery and survival, progression to chronic inflammation is undesirable. Chronic inflammation underlies the pathogenesis of several diseases including diabetes, chronic obstructive pulmonary disorder (COPD), chronic kidney disease, cancer, neurodegenerative and cardiovascular diseases. It has been recognized as the most significant cause of death worldwide, with more than 50% of deaths attributed to the aforementioned underlying pathogenesis [1]. One of the causative factors of inflammation is oxidative stress, which results from the overproduction of reactive oxygen species (ROS) in excessive amounts that cannot all be neutralized by the endogenous antioxidant defense mechanisms [2]. A significant body of evidence supports the link between oxidative stress and inflammation [2,3,4]. High levels of ROS not only damage cellular structures, including lipids, proteins and nucleic acid, but also activate a variety of transcriptional factors, resulting in differential expression of genes involved in inflammatory pathways that ultimately lead to chronic inflammation and can even progress to cancer [3,5,6].

To combat these damaging effects of oxidative stress, cells are equipped with antioxidant defensive mechanisms responsible for the clearance of ROS and maintenance of cellular redox homeostasis. Kelch ECH-associated protein 1 (Keap1)/nuclear factor erythroid 2-related factor 2 (Nrf2)−antioxidant response element (ARE) signaling is a key cytoprotective pathway involved in the induction of phase II detoxification enzymes and therefore protects cells from the accumulation of toxic metabolites [3]. Keap1, a negative regulator of Nrf2 in the cytoplasm, is a cysteine-rich protein (27 cysteine residues) and, upon alkylation of specific Cys residues (usually C151) by electrophiles, induces Nrf2 translocation to the nucleus, where it binds to the ARE and induces the expression of antioxidant genes (*NQO1*, *HO-1*) and suppresses NF-κB-dependent proinflammatory genes (*iNOS*, *COX2*) [3,7,8,9,10]. Hence, the Keap1/Nrf2−ARE pathway is considered a promising therapeutic target for the management of several inflammatory and oxidative-stress-mediated diseases. This is further supported by the fact that bardoxolone methyl, which is an Nrf2 activator, has reached phase III clinical trials for the management of diabetic kidney disease (NCT03550443) [11].

**Figure 1:**
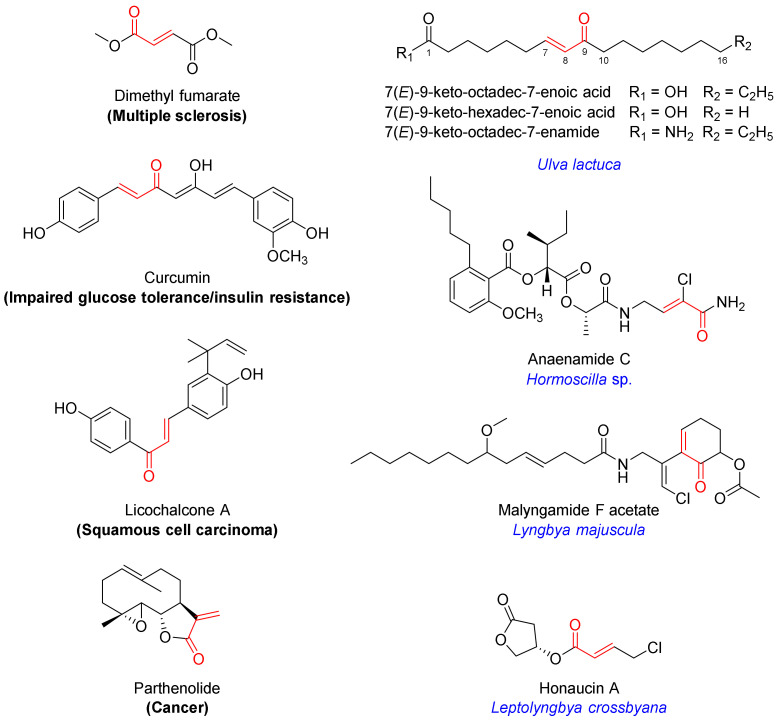
Chemical structures of selected Nrf2 activators bearing an α,*β*-unsaturated carbonyl motif which are either FDA-approved (dimethyl fumarate) or in clinical trials (curcumin, licochalcone A, pathenolide), isolated from algae (monounsaturated fatty acids) or marine cyanobacteria (anaenamide C, malyngamide F acetate, honaucin A).

Among the well-established Nrf2 activators are molecules bearing an α,*β*-unsaturated carbonyl moiety [7,8]. They activate Nrf2 via electrophilic modification of cysteine residues on the Keap1 protein through the 1,4-conjugate addition (Michael) reaction [7,8]. Their chemical reactivity and specific molecular architecture dictate the target profile in the cysteome within and beyond Keap1, producing a net effect. Levels of pleiotropic effects at the target level and downstream signaling contribute to both therapeutic efficacy and toxicity, which has to be taken into consideration. The α,*β*-unsaturated moiety-bearing Nrf2 activator dimethyl fumarate has been approved by the FDA for the management of multiple sclerosis, while several other multi-target drug candidates are in clinical trials for various indications, including curcumin (impaired glucose tolerance and insulin resistance; NCT01052025), licochalcone A (squamous cell carcinoma; NCT03292822) and parthenolide (cancer; NCT00133341) (Figure 1) [7].

Several marine-derived Nrf2 modulators have been reported [12]. In particular, marine algae are known for their cytoprotective effects, and several secondary metabolites have been isolated and characterized as inducers of the Keap1/Nrf2−ARE pathway [13,14]. Marine cyanobacteria are a rich source of structurally diverse secondary metabolites that elicit a wide range of biological activities [15,16,17,18]. Several natural products containing an α,*β*-unsaturated carbonyl system have been isolated from marine benthic cyanobacteria and are reported to induce the activation of Nrf2, including anaenamides C and D (*Hormoscilla* sp.), honaucin A (*Leptolyngbya crossbyana*), and malyngamide F acetate (*Lyngbya majuscula*) (Figure 1) [19,20,21].

**Figure 2:**
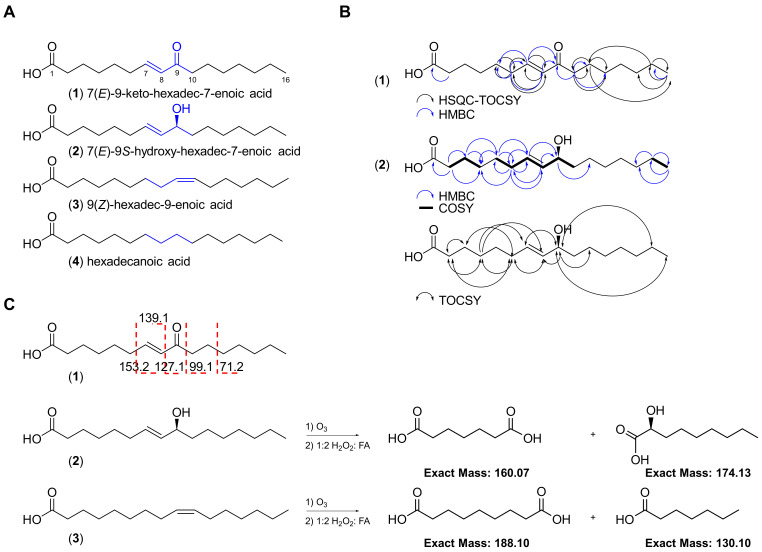
Chemical structures of the four fatty acids (**1**–**4**) isolated from the marine cyanobacterial mat VPFK21-7. (**A**) Key differences in the structures of compounds **1**–**4** are highlighted. (**B**) Key HSQC-TOCSY, HMBC, COSY and TOCSY correlations for compounds **1** and **2**. (**C**) ESI-MS/MS fragmentation and ozonolysis reactions to confirm the position of the double bond in compounds **1**–**3**.

Our efforts to explore field collections of marine cyanobacterial mats from the Florida Keys and Guam have led to the discovery of a monounsaturated fatty acid bearing an α,*β*-unsaturated carbonyl (**1**) in addition to three related analogues (**2**–**4**; Figure 2A). Herein, we describe their isolation, structure elucidation and Nrf2−ARE activity. We report the anti-inflammatory effects of the active compound **1** in lipopolysaccharide (LPS)-stimulated mouse macrophages and captured global transcriptional responses to identify pathways modulated by **1**.

## 2. Results

### 2.1. Isolation and Structure Elucidation

The red marine cyanobacterial mat (VPFK21-7) collected off Delta Shoal in the Florida Keys was freeze-dried and extracted with 1:1 EtOAc−MeOH. The non-polar extract was subsequently partitioned between EtOAc and H_2_O. The EtOAc soluble fraction was then fractionated with silica gel column chromatography using a gradient system starting with 30% EtOAc−Hex and ending with 1:1 EtOAc−MeOH to afford four fractions. The fractions eluting with 30% EtOAc−Hex and EtOAc were further purified by reversed-phase HPLC, resulting in the isolation of four fatty acid type compounds differing in oxidation status and degree of unsaturation (**1**–**4**, Figure 2).

The HRESIMS spectrum of **1** in the negative mode showed an ion peak at *m*/*z* 267.1964 [M − H]^−^, suggesting a molecular formula of C_16_H_28_O_3_ which corresponds to 7(*E*)-9-keto-hexadec-7-enoic acid, a known compound previously isolated from the marine diatom *Skeletonema marinoi* and green alga *Ulva lactuca* [14,22]. Analysis of the ^1^H NMR spectrum acquired in CDCl_3_ revealed the characteristic two olefinic protons H-7 (*δ*_H_ 6.83, *dt*) and H-8 (*δ*_H_ 6.08, *d*) indicative of a Michael acceptor motif present in **1**. Compound **1** possessed the same ^1^H and ^13^C NMR chemical shifts, deduced from the ^1^H NMR and HSQC spectra, to the known compound previously isolated from *Ulva lactuca* (Table 1; Figure 2) [14]. The planar structure was confirmed via HMBC and HSQC-TOCSY and the position of the olefinic double bond was established by selective 1D TOCSY and ESI-MS/MS fragmentation (Figure 1 and Figure S1). The fragmentation of the [M − H]^−^ ion of **1** showed the same fragmentation pattern as **1** isolated from the diatom *Chaetoceros karianus* [23]. A strong fragment ion peak at *m*/*z* 127.1 was evident, which corresponds to the cleavage across C-8/C-9. Compound **1** was also isolated through chemical investigations of four other marine cyanobacteria collected from the Florida Keys and Guam, suggesting a widespread occurrence and potentially important ecological role (see experimental section and Supporting Information).

The HRESIMS spectrum of the optically active compound **2** ([α] _D_^20^ ₊ 23 (*c* 0.01, MeOH)) in the negative mode showed an ion peak at *m*/*z* 269.2119 [M − H]^−^, suggesting a molecular formula of C_16_H_30_O_3_ with two degrees of unsaturation. Analysis of the ^1^H NMR spectrum of **2** and direct comparison with **1** revealed the presence of the two characteristic olefinic proton signals H-7 (*δ*_H_ 5.63, *dt*) and H-8 (*δ*_H_ 5.47, *dd*), with an *E* configuration based on their coupling constant (*J* = 15.3 Hz) and allylic proton H-6 (*δ*_H_ 2.05, *dt*), which were all shifted upfield compared to **1**. Additionally, a new methine signal H-9 (*δ*_H_ 4.06, *dt*) was evident in the ^1^H NMR spectrum of **2**. Taken together, these data suggest that the C-9 keto carbonyl in **1** is replaced by a hydroxy–methine in compound **2**. The proposed structure was further confirmed through analysis of the HMBC data, which showed the presence of only one carbonyl (*δ*_C_ 176.6) corresponding to the carboxylic acid as in **1** but missing keto carbonyl (Table 1 and Table S1; Figure 2). The position of the double bond was confirmed via an ozonolysis reaction followed by oxidative workup to generate two fragments, which were detected by ESIMS in the negative mode (*m*/*z* 159.12 [M − H]^−^ corresponding to the dicarboxylic acid fragment and 173.08 [M − H]^−^ corresponding to the alpha hydroxy carboxylic acid fragment), suggesting the cleavage of alkene at C-7/C-8 (Figure 2C and Figure S4). The configuration at C-9 was determined to be *S* based on a comparison of the optical rotation sign of **2** with the previously reported analogue 8(*E*)-10(*S*)-hydroxy-hexadec-8-enoic acid, which had a positive optical rotation sign [24]. Taken together, the structure of **2** corresponds to 7(*E*)-9*S*-hydroxy-hexadec-7-enoic acid, a known compound previously isolated from the marine diatom *Thalassiosira rotula* [25].

Analysis of the ^1^H NMR spectrum of **3** and direct comparison with **2** revealed the presence of two olefinic protons, H-9 and H-10 (*δ*_H_ 5.34, 2H), and the absence of the methine H-9 (*δ*_H_ 4.06, *dt*) present in **2** (Figure 2). The proposed structure was confirmed with HRESIMS, which gave the expected ion peak that showed 16 mass units of difference compared to **2**, suggesting the lack of one oxygen. The HRESIMS spectrum of **3** in the negative mode showed an ion peak at *m*/*z* 253.2170 [M − H]^−^, suggesting a molecular formula of C_16_H_30_O_2_ with one degree of unsaturation, which corresponds to palmitoleic acid. The position of the double bond was established via an ozonolysis reaction followed by oxidative workup to cleave the alkene and generate a dicarboxylic acid fragment. The fragment was detected by ESIMS in the negative mode, which showed an ion peak at *m*/*z* 187.11 [M − H]^−^, suggesting the cleavage of the alkene at C-9/C-10 (Figure 2C and Figure S4). The *Z* configuration of the double bond was established following a selective homonuclear decoupling of the allylic methylenes (*δ*_H_ 2.04) followed by subsequent measurement of the coupling constant of the olefinic protons (*δ*_H_ 5.34, *J* = 11.1 Hz) and comparison with the coupling constants of commercially available *E*/*Z* isomers of the same compound (Figure S5) [26]. The coupling constants and the ^1^H NMR signals of olefinic protons following homonuclear decoupling were consistent with the published data of isomeric methyl ester derivatives of **3** [26]. Analysis of other proton signals in **3** further confirmed the structural assignments, including the alpha protons H_2_-2 (*δ*_H_ 2.37, *t*, 2H); two allylic methylenes, H_2_-8 and H_2_-11 (*δ*_H_ 2.04, *q*, 4H); one methylene at the beta carbon of the carboxylic acid, H_2_-3 (*δ*_H_ 1.66, *m*, 2H); the methylene chains H_2_-4 to H_2_-7 and H_2_-11 to H_2_-15 (*δ*_H_ 1.29–1.33, *m*, 16H); and one terminal methyl group, H_3_-16 (*δ*_H_ 0.9, *t*, 3H) (Figure 2; Table 1 and Table S1).

Analysis of the ^1^H NMR spectrum of **4** and direct comparison with **3** revealed the absence of the two olefinic protons, H-7 and H-8 (*δ*_H_ 5.37, *m*, 2H), present in **3**. The proposed structure was confirmed by HRESIMS, which gave the expected ion peak that showed 2 mass units of difference to **3**, suggesting the lack of a double bond (Figure 2). The HRESIMS spectrum of **4** in the negative mode showed an ion peak at *m*/*z* 255.2325 [M − H]^−^, suggesting a molecular formula of C_16_H_32_O_2_ with a lower unsaturation number (1), which corresponds to palmitic acid. The structure was deduced via analysis of the ^1^H NMR spectrum, indicating a long-chain fatty acid without branching. The ^1^H NMR spectrum acquired in CDCl_3_ revealed signals indicative of methylene protons at the alpha carbon H_2_-2 (*δ*_H_ 2.37, *t*, 2H), one methylene at the beta carbon of the carboxylic acid H_2_-3 (*δ*_H_ 1.66, *q*, 2H), the methylene chain H_2_-4 to H_2_-15 (*δ*_H_ 1.32, *m*, 12H) and one terminal methyl group, H_3_-16 (*δ*_H_ 0.9, *t*, 3H) (Figure 2; Table 1).

### 2.2. Biological Activity

#### 2.2.1. ARE-Luciferase Reporter Assay

**Figure 3:**
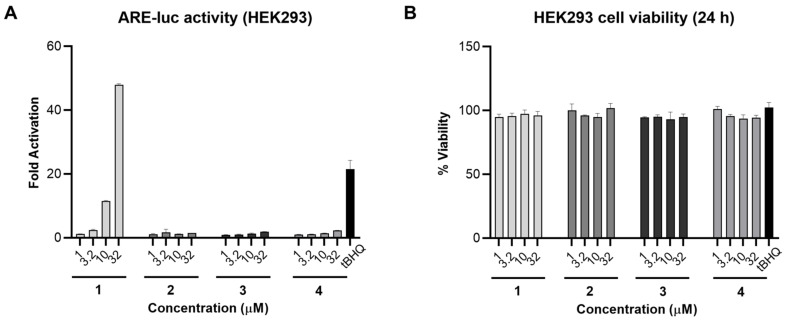
Nrf2/ARE activity for compounds **1**–**4** in HEK293 cells. (**A**) Compound **1** activated the ARE-luc reporter (24 h) in a dose-dependent manner. tBHQ was used as a positive control. (**B**) Cell viability in HEK293 cells after 24 h treatment with **1**–**4** measured by MTT. Data represent mean ± SD (*n* = 3).

As compound **1** was reported previously to be the Nrf2/ARE activator in IMR-32 cells [14], we aimed to investigate the Nrf2/ARE activity of the isolated analogues alongside **1**. The four fatty acids were screened in an ARE-luciferase reporter gene assay for 24 h using stably transfected HEK293 cells. Compound **1**, bearing an α,*β*-unsaturated carbonyl system, induced Nrf2 activity 48- and 11-fold at 32 and 10 µM, respectively, and was slightly active at 3.2 µM. On the other hand, compounds **2**–**4** were not active in this assay (Figure 3A). A cell viability assay was also carried out in parallel under the same conditions and none of the tested compounds were toxic at 32 µM (Figure 3B). Comparing the chemical structures of the fatty acids **1**–**4** with their corresponding ARE-luc activity suggests that the electrophilic Michael acceptor motif is essential for activity. As **1** was the only active compound in this assay, we carried out all subsequent biological studies using **1** that was isolated from VPFK 21-7.

#### 2.2.2. NO Production

**Figure 4:**
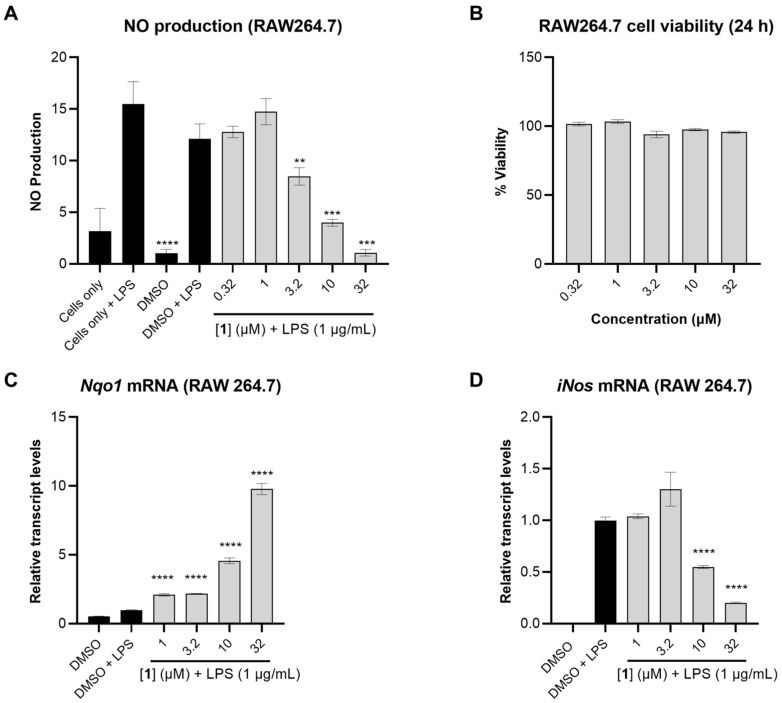
The anti-inflammatory effects of **1** in RAW264.7 cells. (**A**) NO production in RAW264.7 cells measured after 24 h using Griess reagent. The cells were pretreated with **1** at different concentrations for 1 h followed by stimulation with LPS (1 µg/mL). (**B**) Cell viability in RAW264.7 after 24 h treatment with **1** measured using MTT assay. (**C**,**D**) Relative transcript levels of *Nqo1* and *iNos* measured by TaqMan analysis, using actin as endogenous control. RAW264.7 cells were pretreated with compound **1** at different concentrations for 1 h followed by LPS stimulation (1 µg/mL). RNA was extracted following 12 h incubation. Data represent mean ± SD (*n* = 3). The asterisks denote significance of *p* < 0.05 relative to DMSO + LPS using two-tailed unpaired *t*-test (** denotes *p* ≤ 0.01, *** denotes *p* ≤ 0.001, **** denotes *p* ≤ 0.0001).

The anti-inflammatory activity of **1** was subsequently tested by measuring the levels of nitric oxide (NO) in LPS-activated mouse macrophage RAW264.7 cells, a more relevant model for inflammation. In this assay, **1** dose-dependently reduced NO levels up to 3.2 µM (Figure 4A). The cell viability was measured in RAW264.7 cells using MTT, and none of the tested concentrations of **1** were cytotoxic under these conditions (Figure 4B).

#### 2.2.3. Nqo1 and iNos Transcript Levels

To investigate whether the reduction in NO levels caused by **1** was a result of transcriptional regulation, the relative transcript levels of NAD(P)H:quinone oxidoreductase 1 (Nqo1), the mRNA level of a well-known target gene of Nrf2, was measured. Compound **1** dose-dependently induced *Nqo1* transcript levels with pronounced effects observed at 32 and 10 µM (9.8- and 4.6-fold induction, respectively). Concomitantly, **1** suppressed *iNos* transcript levels 5.0- and 2.0-fold at 32 and 10 µM, respectively (Figure 4C,D). *iNos* is an NF-κB target gene which encodes the synthesis of NO. As expected, the suppression of *iNos* transcript levels by **1** translated into reduced levels of nitric oxide in LPS-activated mouse macrophage RAW264.7 cells (Figure 4A).

#### 2.2.4. RNA Sequencing

**Figure 5:**
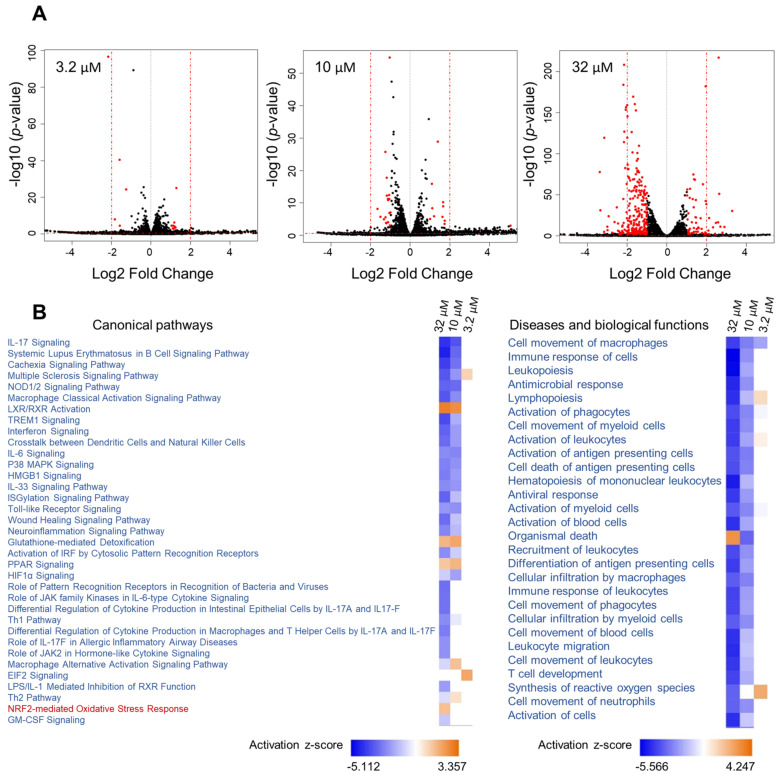
Analysis of RNA sequencing data of **1** at three concentrations. (**A**) Volcano plots of the differentially regulated genes obtained from the RNA sequencing data of **1**, with cutoff values of log2 fold change > 1 and *p*-value < 0.05. (**B**) Heatmap showing selected canonical pathways involved in inflammation and top biological functions of **1** derived from comparison analysis of the three datasets using IPA.

Given the effect of **1** on *Nqo1* and *iNos* mRNA levels linked to two different transcription factors (Nrf2 vs. NF-κB), we aimed to further capture its effects in mouse macrophage RAW264.7 cells on a global level and explore additional pathways that might be modulated. RNA sequencing was performed using three different concentrations of **1** (32, 10 and 3.2 µM), encompassing the entire activity range (Figure 5). This was followed by Ingenuity Pathway Analysis (IPA) of differentially expressed genes using the cutoff criteria of a 1.5-fold change (exp log ratio 0.586) and *p*-value of 0.05 to focus on biologically and statistically significant changes. Using these criteria, a total of 671 genes (at 32 µM), 325 genes (at 10 µM) and 130 genes (at 3.2 µM) were identified as differentially expressed (exp log ratio 0.586 and *p*-value of 0.05).

At the concentration where **1** showed the most potent activity (32 µM, Figure 4), the dataset was highly enriched with genes involved in inflammatory pathways, and at 10 µM similar results were noted but with differences in z-scores and *p*-values (Figure 5B). At 3.2 µM, where the compound showed only marginal activity (Figure 4), these pathways were not enriched (Figure 5B).

Analysis of RNA sequencing data of **1** at 32 µM and 10 µM revealed immunological and inflammatory diseases, immune cell trafficking and inflammatory response to be among the top hits in the IPA list of diseases and functions. Additionally, functions related to lipid metabolism and free radical scavenging (synthesis of reactive oxygen species) were also detected. The analysis supports the beneficial functions of **1**, as the activation status of some of the diseases and functions underlying the aforementioned categories were predicted to be decreased based on the activation z-scores. As expected, the Nrf2-mediated oxidative stress response and glutathione-mediated detoxification pathways were evident in the analysis with positive z-scores (Table 2). Additionally, several other canonical pathways with negative z-scores, which strongly correlate with inflammation, were detected (Table 2).

Hypercytokinemia/hyperchemokinemia and pathogen-induced cytokine storm were the top two canonical pathways that were downregulated. Next, pattern recognition receptor (PRR) signaling was identified as downregulated; this receptor recognizes specific molecules on the surface of pathogens and induces the innate immune response, which in turn activates downstream signaling pathways that activate transcriptional responses and trigger the expression and release of pro-inflammatory genes to initiate host defense [27]. Also downregulated were NOD-like receptors, members of PRRs which activate the innate immune system in response to cellular stress and injury and therefore are associated with a wide range of inflammatory conditions [28]. In addition to NF-κB and MAPK, NOD1/2 is involved in the activation of interferon (IFN) signaling, which was strongly attenuated based on our analysis [29]. IL-17 signaling, a critical pathway involved in the pathogenesis of several inflammatory and autoimmune diseases, was also downregulated [30]. In these conditions, IL-17 induces the sustained production of inflammatory cytokines and chemokines, including IL-1, IL-6, IL-1β, TNF, CCL2 and CSF, as well as matrix metalloproteinases such as MMP3 and MMP9, all of which were detected and found to be downregulated in response to **1** [31]. Additional players in the pathogenesis of inflammatory and autoimmune diseases that were downregulated were the high-mobility group box 1 (HMGB1) and p38 mitogen-activated protein kinases (MAPK) signaling [32,33]. Interestingly, peroxisome proliferator-activated receptor (PPAR) signaling weas upregulated. PPARs are ligand-activated transcriptional regulators involved in lipid metabolism and therefore implicated in the regulation of several cellular processes [34]. PPARs heterodimerize with retinoic X receptors (RXR)—which were also upregulated—and upon ligand (unsaturated fatty acids) binding they regulate the expression of downstream target genes [34].

**Figure 6:**
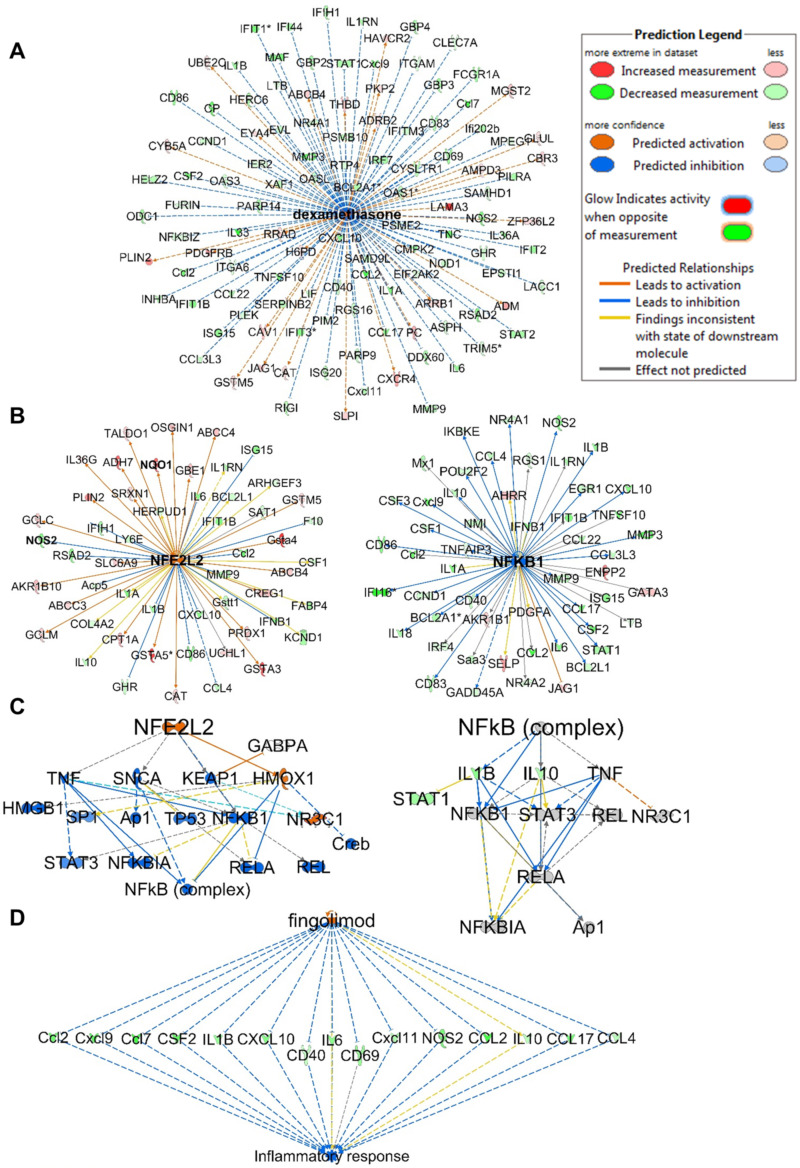
IPA analysis of RNA sequencing data at 32 µM. (**A**) Upstream regulator network of dexamethasone showing all genes with measurement directions consistent with dexamethasone’s predicted activation state. (**B**) Upstream regulator networks of Nrf2 (NFE2L2) and NF-κB showing the modulated target genes in response to **1**. (**C**) Mechanistic networks of Nrf2 (NFE2L2) and NF-κB demonstrating the crosstalk between the two pathways via HMOX1 activation. (**D**) Regulator effect network of the Nrf2 activator fingolimod, predicted to be activated as it downregulates the same set of genes that are downregulated in the dataset in response to **1**. The asterisks on some genes indicate that multiple identifiers in the dataset file map to a single gene.

The top upstream regulator identified in the analysis was lipopolysaccharide (LPS), which was predicted to be inhibited with activation z-scores of −11.621 and −5.491 at 32 and 10 µM, respectively (Figure S6). Interestingly, the upstream analysis identified dexamethasone, which was predicted to be activated with positive z-scores of 4.210 and 5.299 at 32 and 10 µM, respectively. The analysis identified 116 genes out of 202 (at 32 µM) in the dataset with measurement directions consistent with its activation, suggesting that compound **1** functions similarly to dexamethasone (Figure 6A). Dexamethasone is a steroidal drug known for its anti-inflammatory activity. Several studies supported the effect of dexamethasone in inhibiting *iNos* expression and NO production including LPS-treated macrophages [35,36,37,38,39,40,41,42,43]. Dexamethasone was also reported to inhibit TNFα secretion and LPS-induced activation of p38 MAPK signaling. Furthermore, a study supported its effects of inhibiting *IL-1β* expression via inhibition of NF-κB/Rel and AP1 in LPS-stimulated RAW264.7 cells [36,44]. The upstream analysis identified 11 genes (downregulated: *CCL2, NOS2, IL-6, IL-1β, MMP9, CCL4, IL-10, CCL3L3, CCND1, BCL2L1*; upregulated: *GCLM*) in the dataset with the same measurement direction as the Nrf2 activator bardoxolone methyl, a natural product-derived triterpenoid (z-score 3.251; *p*-value 5.82 × 10^−11^) which has reached phase III clinical trials for the treatment of diabetic kidney disease [11]. Nrf2 (NFE2L2) was detected as an upstream regulator and predicted to be activated (z-score 4.198), with 40 out of 53 genes having a measurement direction consistent with its activation (Figure 6B). The analysis also identified NF-κB as an upstream regulator that is predicted to be inhibited (z-score −4.170) based on 30 out of 52 genes having a measurement direction consistent with its inhibition, including *IL-6, IL-10, IL-1β, NOS, MMP3* and *MMP9*, which were all significantly downregulated (Figure 6B). In line with the reported crosstalk between the Nrf2 and NF-κB pathways [45,46], the mechanistic network of the upstream regulator Nrf2 shows and predicts HMOX1 activation as a key player mediating the crosstalk through the reported inhibition of proinflammatory genes of NF-κB (Figure 6C) [47].

Analysis of regulator effect networks at 32 µM identified the known ARE activators fingolimod, resveratrol and curcumin to have similar effects as **1** [6,48]. These networks connect the upstream regulator through differentially regulated genes in the dataset to a relevant phenotype (Figure 6D). Fingolimod, a natural product-derived drug for the treatment of multiple sclerosis, is known for its immunosuppressive and neuroprotective effects and is currently in phase 2 clinical trials for inflammation (NCT04675762). Studies have supported the neuroprotective effects of fingolimod in multiple sclerosis, which are mediated through downregulation of IL-17 signaling and inhibition of NF-κB translocation and NO production in astrocytes [48,49,50]. Interestingly, relapsed multiple sclerosis appeared in the analysis list of diseases and was predicted to be decreased based on the activation z-score −2.058 (*p*-value 2.28 × 10^−12^) with 22 genes involved. Also, multiple sclerosis signaling was one of the top canonical pathways identified in the analysis and was predicted to be downregulated. Resveratrol, a plant-derived polyphenolic compound known for its antioxidant and anti-inflammatory activities, also appeared in the analysis, modulating a set of genes with similar measurement direction as **1** (Figure S7). Resveratrol was reported to decrease the production of *IL-6*, *TNF-α* and *IL-17* expression in HTLV-1-infected T cells, as well as reduce NO production and inhibit *iNos* expression in LPS-stimulated RAW264.7 cells [51].

## 3. Discussion

Marine cyanobacteria are prolific producers of bioactive secondary metabolites with diverse structures, including polyketides, modified peptides and fatty acid derivatives such as fatty acid amides [15,16,17,18]. Here, we report for the first time the discovery and isolation of a cyanobacterial C16 monounsaturated fatty acid (**1**) and its hydroxy-containing analogue (**2**), which were previously reported in marine diatoms and a green alga, in addition to two other structurally related analogues (**3**–**4**), all differing in their oxidation status. Compound **1**, characterized by the presence of a conjugated enone motif, has been previously isolated from the green alga *Ulva lactuca* and was reported to induce Nrf2/ARE activity in IMR-32 cells [14]. Also, it is an isomer of the anti-inflammatory (*E*)-9-oxohexadec-10-enoic acid isolated from the red alga *Gracilaria verrucosa*, which was reported to inhibit the production of NO, IL-6 and TNFα in LPS-stimulated RAW264.7 cells [24]. Furthermore, **1** and its isomer were also co-isolated from the diatom *Chaetoceros karianus*, which both exhibited a dual agonist activity towards human PPARα and PPARγ [23]. The co-existence of both the keto- and hydroxy-monounsaturated fatty acids has been reported previously, as **1** and **2** were isolated from the marine diatom *Skeletonema marinoi* [22]. C18 analogues of **1** and **2**, namely (*E*)-11-oxo-octadeca-12-enoic acid and (*E*)-11-hydroxy-octadeca-12-enoic acid, were isolated from a marine green alga, *Ulva fasciata* [52]. Furthermore, the methyl ester derivatives of **1** and **2** have been reported from the marine diatom *Thalassiosira rotula*, in which the extract was methylated prior to purification of both derivatives [25].

In this study, we also report the discovery of **1** from cyanobacterial samples collected from five distinct geographical locations in Guam and Florida Keys. The widespread distribution of these secondary metabolites across different organisms and geographical locations highlights their evolutionary importance, which prompted us to investigate their bioactivity to better understand their pervasiveness in the marine environment. C16 acid (**1**) and its derivatives 7(*E*)-9-keto-octadec-7-enoic acid (C18 acid) and 7(*E*)-9-keto-octadec-7-enamide (C18 amide) from *Ulva lactuca* demonstrated Nrf2/ARE activity in IMR-32 human neuroblastoma, a widely used cellular model for oxidative stress, with the highest ARE activation induced by the C18 acid derivative of **1** with no observed effects on viability [14]. The C18 acid also induced many ARE-regulated antioxidant genes in vitro, including *Nqo1*, and hence highlighting the potential of these unsaturated fatty acids for chemoprevention to protect from cancer and other oxidative-stress-mediated diseases. Due to the established crosstalk between oxidative stress and inflammation, we investigated the anti-inflammatory potential of **1** in RAW264.7 mouse macrophage cells, a relevant cellular model for inflammation. Compound **1** suppressed the LPS-induced transcript levels of the NF-κB target gene *iNos*, which translated into reduced production of NO. This data inversely correlated with *Nqo1* transcript levels, which showed a strong dose-dependent induction in response to **1**, suggesting that the anti-inflammatory activity of **1** is mediated through ARE activation. Our analysis of LPS-induced global changes in transcript levels following treatment with **1** identified many additional canonical pathways and regulator networks linked to inflammation, immune response and related diseases, particularly multiple sclerosis. Recent evidence highlights the role of Nrf2 as a regulator of innate immune response via direct and indirect interactions with major components and signaling pathways of the immune system, including Toll-like receptor signaling, the NF-κB pathway and the type-1 interferon system [53], all of which were found to be downregulated in our IPA analysis. Furthermore, Nrf2 has been reported to be involved in the regulation of immune cell recruitment, as well as cytokine release and transcriptional activation of proinflammatory cytokines such as IL-6 and IL-1β, which were downregulated in response to **1** in LPS-stimulated macrophages.

Oxidative stress and high levels of ROS have been reported to underlie the pathogenesis of several neurodegenerative disorders, including multiple sclerosis. In multiple sclerosis, macrophages and microglia activation contribute to increased levels of ROS, dysregulated immunity and enhanced inflammatory response, ultimately resulting in mitochondrial dysfunction, neuroinflammation, demyelination and axonal degeneration, key features of the disease [54]. High levels of ROS in multiple sclerosis were reported to activate immune cells to induce kinases and redox-sensitive transcription factors, including MAPKs, AP1 and NF-κB. NF-κB activation in turn upregulates the expression of several genes implicated in the pathogenesis of multiple sclerosis, such as *TNFα*, *iNos* and *IL1α*/*IL1β* [55]. Increased levels of TNFα, IL-6 and IL-10 were evident in the blood of an LPS-treated animal model of multiple sclerosis [54]. Interestingly, our transcriptomic analysis revealed downregulation of all these markers in response to **1**. An increasing body of evidence supports the involvement of Nrf2 in the pathogenesis of multiple sclerosis in particular, where loss of Nrf2 resulted in a rapid onset and more severe clinical course of the disease [54]. The role of Nrf2 activation as a potential therapeutic target was further supported following the FDA approval of the Nrf2 activator dimethyl fumarate for the treatment of remitting–relapsing multiple sclerosis [54,55,56]. The drug was shown to induce *Nqo1* expression levels in patients’ blood following 4–6 weeks’ treatment, with more patients likely to have undetected disease activity a year later [54,57]. In a mouse model of multiple sclerosis, dimethyl fumarate treatment reduced the severity of symptoms and preserved myelination compared to Nrf2^−/−^ mice [55,56]. Interestingly, our transcriptomic analysis identified multiple sclerosis signaling as among the top five canonical pathways that were downregulated, in addition to the predicted inhibition of relapsed multiple sclerosis in the list of diseases. Also, the identification of the multiple sclerosis drug fingolimod in regulator effect networks, in addition to resveratrol and curcumin, which were all preclinically investigated as Nrf2 activators in experimental models of multiple sclerosis, further supports the role of the Nrf2-ARE signaling pathway activation in combating the hallmark features of the disease and highlights the potential beneficial applications of **1** [55].

## 4. Materials and Methods

### 4.1. Biological Material

Samples of VPFK21-7, a red cyanobacterial mat, were collected from Delta Shoal in the Florida Keys on 24 September 2021. While the taxonomy of this cyanobacterium has not been confirmed, the fine filaments were only ~5 µm in width and were fairly uniform, suggesting mostly one filament type in the sample. Samples of the cyanobacterium DRTO-73, which correspond microscopically with *Leptolyngbya* sp., were collected from Loggerhead Key, Florida on 21 May 2013 [58]. Samples of the cyanobacterium VPG 18-67 red leathery mat were collected from Tanguisson Reef flat in Guam on 7 April 2018.

Two other cyanobacteria, HL-CN2011-062 (DRTO-46) and HL-CN2015-131 (DRTO-89), were collected from Fort Jefferson on 24 May 2011 and Loggerhead Key on 9 May 2015, respectively. This is a diverse group of benthic filamentous cyanobacteria. Voucher specimens have been retained at the Smithsonian Marine Station for all samples.

### 4.2. General Experimental Procedures

^1^H and 2D NMR spectra for **1** (0.4 mg) and **2** (0.3 mg) in CDCl_3_ were recorded on a Bruker 800-MHz/63 mm Avance III Spectrometer (Bruker Biospin Corporation, Billerica, MA, USA) and Bruker Avance Neo-600-MHz Spectrometer using a 1.7 mm tube, respectively. ^1^H NMR spectra for **3** (0.2 mg) and **4** (0.8 mg) in CDCl_3_ were recorded on a Cryo 600-MHz/54 mm Bruker Avance III HD. The spectra were referenced using the residual solvent signal (δ_H_/_C_ 7.26/77.0 (CDCl_3_)). The HRESIMS data were obtained in the negative mode using the high-resolution LC Q-Exactive orbitrap mass spectrometer (ThermoSci, Waltham, MA, USA) equipped with the APCI/ESI multimode ion source detector. The LRESIMS data were acquired in the negative mode using the TSQ Altis plus triple quadrupole mass spectrometer equipped with Vanquish LC system (ThermoSci). The optical rotation was measured using a JASCO P-2000 polarimeter.

### 4.3. Extraction and Isolation

Samples of the VP FK21-7 red mat cyanobacterium were freeze-dried and subjected to non-polar extraction using EtOAc−MeOH (1:1) and polar extraction using 30% aq EtOH. The non-polar extract (2724 mg) was partitioned between EtOAc and H_2_O. The EtOAc fraction (80.5 mg) was concentrated and subjected to silica gel chromatography using the following gradient: (30% EtOAc−Hex, 100% EtOAc, 10% MeOH−EtOAc, 1:1 EtOAc−MeOH, and finally 100% MeOH). The fraction that eluted with 100% EtOAc (12 mg) was purified by semipreparative reversed-phase HPLC [Synergi Hydro 4u-RP, 250 × 10.0 mm; flow rate, 2.0 mL/min; PDA detection 200–800 nm] using a linear MeOH−H_2_O gradient (20–100% MeOH over 15 min, 100% MeOH for 20 min) to afford **1** and **2** in a 1:1 mixture (1.5 mg) eluting at *t*_R_ 21.6 min. The mixture was further purified by HPLC using an analytical column [Synergi 4u Hydro-RP 80A, 150 × 4.60 mm, 4 micron; flow rate 0.5 mL/min, PDA detection 200–800 nm] with a linear ACN−H_2_O gradient (20–100% ACN for 10 min, 100% ACN for 15 min) to afford **1** (0.4 mg), eluting at *t*_R_ 12.2 min, and **2** (0.3 mg), eluting at *t*_R_ 12.0 min. The Si fraction that eluted with 30% EtOAc−Hex (34.3 mg) was partially purified (11.6 mg) by semipreparative reversed-phase HPLC [Synergi Hydro 4u-RP, 250 × 10.0 mm; flow rate, 2.0 mL/min; PDA detection 200−800 nm] using a linear MeOH−H_2_O gradient (20–100% MeOH over 15 min, 100% MeOH for 20 min) to afford **3** (0.8 mg), eluting at *t*_R_ 26.5 min, and **4** (0.8 mg), eluting at *t*_R_ 28.3 min. Compound **3** was repurified by HPLC using analytical column [Synergi 4u Hydro-RP 80A, 150 × 4.60 mm, 4 micron; flow rate 0.5 mL/min, PDA detection 200–800 nm] using a linear ACN−H_2_O gradient (20–100% ACN for 10 min, 100% ACN for 15 min) to afford **3** (0.2 mg) eluting at *t*_R_ 17.3 min.

7(*E*)-9-Keto-hexadec-7-enoic acid (**1**): colorless, amorphous powder; ^1^H and 2D NMR data (CDCl_3_), Table 1; HRESIMS *m*/*z* 267.1964 [M − H]^−^ (calcd. for C_16_H_27_O_3_, 268.3970).

7(*E*)-9*S*-Hydroxy-hexadec-7-enoic acid (**2**): colorless, amorphous powder; [α] _D_^20^ ₊ 23 (*c* 0.01, MeOH); ^1^H and 2D NMR data (CDCl_3_), Table 1 and Table S1; HRESIMS *m*/*z* 269.2119 [M − H]^−^ (calcd. for C_16_H_29_O_3_, 270.4130).

9(*Z*)-Hexadec-9-enoic acid (**3**): colorless, amorphous powder; ^1^H NMR and HSQC data (CDCl_3_), Table 1 and Table S1; HRESIMS *m*/*z* 253.2170 [M − H]^−^ (calcd. for C_16_H_29_O_2_, 254.4140).

Hexadecanoic acid (**4**): colorless, amorphous powder; ^1^H NMR data (CDCl_3_), Table 1; HRESIMS *m*/*z* 255.2325 [M − H]^−^ (calcd. for C_16_H_31_O_2_, 256.4300).

#### Isolation of Compound **1** from Other Cyanobacteria Samples

Samples of the filamentous cyanobacterium DRTO-73 collected from Loggerhead Key, Florida were freeze-dried, extracted and fractionated as described previously [58]. The 20% *i*PrOH/CH_2_Cl_2_ was purified by reversed-phase HPLC [Synergi Hydro 4u-RP, 250 × 10.0 mm; flow rate, 2.0 mL/min; PDA detection 200–800 nm] using a linear MeOH−H_2_O gradient (60–100% MeOH over 10 min, 100% MeOH for 20 min) to afford **1** (0.6 mg) and which was repurified by HPLC [Synergi 4u Hydro-RP 80A, 150 × 4.60 mm, 4 micron; flow rate 0.5 mL/min, PDA detection 200–800 nm] using a linear ACN−H_2_O gradient (20–100% ACN for 10 min, 100% ACN for 15 min) to afford **1** (0.1 mg) eluting at *t*_R_ 12.2 min.

The freeze-dried material of the cyanobacterium VPG18-67 red leathery mat collected in Guam (73.35 g) was subjected to non-polar extraction using EtOAc−MeOH (1:1) and polar extraction using EtOH−H_2_O (1:1). The non-polar extract (2.4 g) was partitioned between hexanes and 80% MeOH in H_2_O. The methanolic phase was dried and further partitioned between EtOAc and H_2_O. The EtOAc layer was concentrated and subjected to silica gel chromatography by using hexane and increasing gradients of EtOAc (10, 20, 40, 60, 80%), 100% EtOAc, MeOH and increasing gradients of EtOAc (20, 40, 60, 80%), and finally with 100% MeOH. The 60% EtOAc/hexane Si fraction was purified by reversed-phase HPLC [Luna C18, 250 × 4.60 mm, 4 micron; flow rate 0.8 mL/min, PDA detection 200–800 nm] using a linear ACN−H_2_O gradient (10–95% ACN for 30 min, 95% ACN for 10 min) to afford **1** (0.4 mg) eluting at *t*_R_ 28.3 min.

The freeze-dried material (20.56 g) of the cyanobacterium HL-CN2011-062 (DRTO-46) collected from Fort Jefferson was extracted and partitioned as described for the sample VPG18-67. The EtOAc-partitioned fraction (60.47 mg) was subjected to silica gel chromatography by using CH_2_Cl_2_ and increasing gradients of *i*PrOH (2, 4, 6, 8, 10, 20, 40, 60, 80%), 100% iPrOH, and finally with 100% MeOH. The fraction that was eluted with 2 and 4% *i*PrOH/CH_2_Cl_2_ contained compound **1** based on the characteristic downfield signals in the ^1^H NMR spectrum characteristic of the Michael acceptor motif. A total of 4 mg was then purified by reversed-phase HPLC [Synergi 4u Hydro-RP 80A, 150 × 4.60 mm, 4 micron; flow rate 1 mL/min, PDA detection 200–800 nm] using a linear ACN−H_2_O gradient (15–100% ACN for 25 min, 100% ACN for 10 min) to afford **1** (0.4 mg) eluting at *t*_R_ 20.1 min.

The freeze-dried material (2.88 g) of the cyanobacterium HL-CN2015-131 (DRTO-89) collected from Loggerhead Key was extracted, partitioned and fractionated as described for HL-CN2011-062. The fraction that was eluted with 6 and 8% *i*PrOH/CH_2_Cl_2_ contained compound **1** based on the characteristic downfield signals in the ^1^H NMR spectrum characteristic of the Michael acceptor motif. A total of 4.63 mg was then purified by reversed-phase HPLC using the same column and method described for HL-CN2011-062 to afford 0.2 mg of **1**.

### 4.4. Ozonolysis and Oxidative Workup

Compounds **2** and **3** (50 µg each) were dissolved in 3 mL of CH_2_Cl_2_ followed by bubbling of ozone into the solution at −78 °C for 20 min. The solution was then dried, and the residues were resuspended in H_2_O_2_−HCOOH (1:2) for 20 min at 70 °C. The resulting mixture was subsequently evaporated and subjected to ESIMS analysis in the negative mode to detect the generated dicarboxylic acid fragments.

### 4.5. Cell Culture

HEK293 ARE-luc cells (SL-0042-NP; Signosis) (human embryonic kidney cells stably transfected with firefly luciferase reporter gene) and RAW264.7 cells (ATCC) (mouse macrophagic cells) were cultured using Dulbecco’s Modified Eagle’s Medium (DMEM; Invitrogen) supplemented with 10% fetal bovine serum (FBS; Sigma, St. Louis, MO, USA), 1% antibiotic–antimycotic (Invitrogen, Waltham, MA, USA) and maintained at 37 °C humidified air and 5% CO_2_.

### 4.6. ARE-Luciferase Reporter Assay

HEK293 ARE-luc cells (10,000 cells/well) were seeded in 96-well plates (Costar 96-well white solid plates; COS3917) and incubated overnight to attach. The cells were then treated with different concentrations of compounds, positive control (tBHQ, 10 µM) and solvent control (DMSO; 0.5% *v*/*v*). Following 24 h incubation, luciferase activity was detected using BriteLite detection reagent (PerkinElmer, Waltham, MA, USA) following the manufacturer’s instructions. The luminescence was read using the Envision plate reader (PerkinElmer). Cell viability assay was performed in parallel under the same conditions and time points.

### 4.7. NO Assay

RAW264.7 cells (20,000 cells/well) were seeded in 96-well plates and allowed to attach overnight. The cells were then pretreated for 1 h with different concentrations of compounds **1** and **2**, and solvent control (DMSO; 0.5% *v*/*v*) followed by stimulation with 1 µg/mL LPS. Non-stimulated cells (no LPS) were also tested simultaneously. Following 24 h incubation, the production of NO in the cell supernatant was assessed by measuring the concentration of nitrile, which is an oxidative product of NO. Briefly, 50 µL of culture supernatant was mixed with 50 µL of Griess reagent (Promega) according to the manufacturer’s instructions. The absorbance was measured at 540 nm using a SpectraMax M5 plate reader (Molecular Devices, SAN Jose, CA, USA). NO production was calculated based on a standard reference curve generated for fresh nitrite standard solution. Cell viability assay was performed in parallel under the same conditions and time points.

### 4.8. Cell Viability Assay

HEK293 ARE-luc cells (10,000 cells/well) and RAW264.7 cells (20,000 cells/well) were seeded in 96-well plates and allowed to attach overnight. The cells were then treated with different concentrations of compounds, positive control (tBHQ, 10 µM) and solvent control (DMSO; 0.5% *v*/*v*). Following 24 h incubation, cell viability was measured using 3-(4,5-dimethylthiazol-2-yl)-2,5-diphenyltetrazoliumbromide according to the manufacturer’s instructions (Promega, Madison, WI, USA).

### 4.9. RNA Extraction and RT-qPCR Analysis of Nqo1 and iNos Transcript Levels in RAW264.7 Cells

RAW264.7 cells (250,000 cells/well) were seeded in 6-well plates and allowed to attach overnight. After overnight incubation, the media in each well were replaced with fresh media prior to treatment with **1** at 32, 10, 3.2 and 1 µM and solvent control (0.5% DMSO) for 1 h. After 1 h, the cells were stimulated with1 µg/mL LPS. Non-stimulated cells (no LPS) were also tested simultaneously. After 12 h incubation, RNA was isolated using an RNeasy mini kit (Qiagen, Hilden, Germany) according to the manufacturer’s protocol. cDNA synthesis was carried out using SuperScript II reverse transcriptase and oligo (dT) (Invitrogen). qPCR was carried out after reverse transcription on a reaction solution (25 μL) prepared using a 1 μL aliquot of cDNA, 12.5 μL of TaqMan gene expression assay mix, 1.25 μL of 20× TaqMan gene expression assay mix and 10.25 μL of RNase-free water. The qPCR experiment was performed using an ABI 7300 sequence detection system. The thermocycler program used was 2 min at 50 °C, 10 min at 95 °C, 15 s at 95 °C (40 cycles) and 1 min at 60 °C. iNos (Mm00440502_m1) and NQO1 (Mm01253561_m1) were used as target genes and mouse ACTB (#4352933, Applied Biosystems, Waltham, MA, USA) was used as endogenous control.

### 4.10. Illumina Sequencing Library Construction 

RNA samples were measured by the QUBIT fluorescent method (Invitrogen) and Agilent Bioanalyzer. A total of 500 ng high-quality total RNA with a RIN of 10 was used for library construction using the reagents provided in the NEBNext Poly(A) mRNA Magnetic Isolation Module (New England Biolabs, Ipswich, MA, USA, catalog # E7490) and the NEBNext Ultra II Directional RNA Library Prep Kit (New England Biolabs, catalog # E7760) according to the manufacturer’s user guide. Briefly, 500 ng of total RNA was used for mRNA isolated using the NEBNext Poly(A) mRNA Magnetic Isolation Module (New England Biolabs, catalog # E7490). Then, the poly A-enriched RNA was fragmented in a NEBNext First Strand Synthesis Buffer via incubation at 94 °C for the desired time. This step was followed by first-strand cDNA synthesis using reverse transcriptase and random hexamer primer. Synthesis of ds cDNA was conducted using the 2nd strand master mix provided in the kit, followed by end repair and dA tailing. At this point, Illumina adaptors were ligated to the sample. Finally, the library was amplified, followed by purification with AMPure beads (Beckman Coulter, Brea, CA, USA, catalog # A63881). The library size and mass were assessed by analysis in the Agilent TapeStation using a High Sensitivity DNA1000 Screen Tape. Typically, a 250–900 library peak was observed, with the highest peak at ~420 bp. Barcoded libraries were pooled equimolarly for sequencing simultaneously for NavaSeq 6000 S4 2 × 150 cycles run as described below. RNA-seq library performed at UF ICBR Gene Expression Core (https://biotech.ufl.edu/gene-expression-genotyping/, accessed on 26 May 2023, RRID:SCR_019145).

### 4.11. Illumina NovaSeq6000 Sequencing

Normalized libraries were submitted to the “Free Adapter Blocking Reagent” protocol (FAB, Cat# 20024145) to minimize the presence of adaptor dimers and index hopping rates. The library pool was diluted to 0.8 nM and sequenced on one S4 flow cell lane (2 × 150 cycles) of the Illumina NovaSeq6000. The instrument’s computer utilized the NovaSeq Control Software v1.6. Cluster and SBS consumables were v1.5. The final loading concentration of the library was 120 pM with 1% PhiX spike-in control. One lane generated 2.5–3 billion paired-end reads (~950 Gb) with an average Q30% ≥ 92.5% and Cluster PF = 85.4%. FastQ files were generated using the BCL2fastQ function in the Illumina BaseSpace portal. The Illumina NovaSeq 6000 was used to sequence the libraries for 2 × 150 cycles. Sequencing was performed with ICBR NextGen Sequencing (https://biotech.ufl.edu/next-gen-dna/, RRID:SCR_019152, accessed on 26 May 2023). The data were deposited with the GEO accession number GSE240402.

## 5. Conclusions

In conclusion, we report the discovery of a C16 monounsaturated fatty acid containing an α,*β*-unsaturated carbonyl (**1**) for the first time from a benthic marine cyanobacterial mat, in addition to three structurally related analogues (**2**–**4**). These compounds were previously known from diatoms and algae and may play an important ecological role. The α,*β*-unsaturated carbonyl motif appeared to be critical for their Nrf2−ARE activity. The relationship of **1** and **2** suggests that oxidation reduction or vice versa can serve as an on/off switch for antioxidant and anti-inflammatory activity. Given the established crosstalk between the Nrf2 and NF-κB pathways, we evaluated the anti-inflammatory potential of **1** in LPS-stimulated mouse macrophages, reducing NO production through a transcriptional mechanism by lowering *iNos* transcript levels. We also demonstrated the effect of **1** on global transcript changes induced by LPS via RNA sequencing and identified additional inflammatory pathways besides the Keap1/Nrf2−ARE pathway modulated by **1**. Transcriptomic analysis identified many additional canonical pathways and regulatory networks linked to inflammation and immune function and related diseases, particularly multiple sclerosis, for which a Nrf2 activator (dimethyl fumarate) is FDA-approved. Our study raises the question of whether therapeutic or preventive effects can be achieved with such oxidized fatty acids. The diversity of organisms producing the compounds allows us to target those that are easiest to grow. While we studied the effects of **1** from a marine cyanobacterium, a previous isolation from the dietary seaweed *Ulva lactuca*, which is easily amenable to culturing, suggests that therapeutic properties could be retrieved through diet or supplementation. This class of α,*β*-unsaturated carbonyl-containing fatty acids could also serve as chemical probes to aid in understanding the molecular basis underlying several inflammatory and oxidative-stress mediated diseases, where the cytoprotective Keap1/Nrf2−ARE pathway serves as a target.

## Figures and Tables

**Table 1:** NMR spectral data for the isolated fatty-acid-type compounds (**1**–**4**) in CDCl_3_.

C/H No.	1		2		3	4
	*δ*_H_ (*J* in Hz)	*δ*_C_ ^a^, mult ^b^	*δ*_H_ (*J* in Hz)	*δ*_C_ ^a^, mult ^b^	*δ*_H_ (*J* in Hz)	*δ*_H_ (*J* in Hz)
OH	−	−	−	−	−	−
1	−	177.2, qC	−	176.6, qC	−	−
2	2.35, t (7.4)	33.7, CH_2_	2.37, t (7.4)	33.5, CH_2_	2.37, t (7.4)	2.37, t (7.4)
3	1.65, m	24.8, CH_2_	1.67, m	24.5, CH_2_	1.67, m	1.67, m
4	1.31, m	31.7, CH_2_	1.37, m	28.7, CH_2_	1.32, m	1.28, m
5	1.49, m	27.9, CH_2_	1.40, m	28.7, CH_2_	1.32, m	1.28, m
6	2.21, dt (7.1, 6.9)	32.3, CH_2_	2.05, dt (7.1, 6.9)	32.1, CH_2_	1.32, m	1.28, m
7	6.83, dt (15.3, 6.9)	147.1, CH	5.63, dt (15.3, 6.9)	132.1, CH	1.32, m	1.28, m
8	6.11, dd (15.3, 7.1)	130.3, CH	5.47, dd (15.3, 7.1)	133.2, CH	2.02, dt (6.9, 6.3)	1.28, m
9	−	201.0, qC	4.06, dt (7.1, 6.8)	73.3, CH	5.34, m	1.28, m
9-OH			−	−	−	−
10	2.54, m	40.2, CH_2_	1.57, 1.49, m	37.3, CH_2_	5.34, m	1.28, m
11	1.62, m	24.2, CH_2_	1.33, m	25.4, CH_2_	2.02, dt (6.9, 6.3)	1.28, m
12, 13	1.37, m	28.7, CH_2_	1.30, m	29.1, CH_2_	1.32, m	1.28, m
14	1.37, m	28.7, CH_2_	1.30, m	31.9, CH_2_	1.32, m	1.28, m
15	1.32, m	22.5, CH_2_	1.31, m	22.5, CH_2_	1.32, m	1.28, m
16	0.9, t (7.0)	14.1, CH_3_	0.9, t (7.0)	14.1, CH_3_	0.9, t (7.0)	0.9, t (7.0)

^a 13^C values were deduced from HSQC and HMBC spectra. ^b^ Multiplicity derived from the HSQC spectrum.

**Table 2:** Selected immune and inflammatory canonical pathways modulated by **1** at 32 µM based on RNA-seq and IPA.

Canonical Pathway	Activation z-Score	*p*-Value	No. of Molecules	Selected Genes
Role of hypercytokinemia/hyperchemokinemia in the pathogenesis of Influenza	−5.112	4.85 × 10^−27^	30	*IL33*, *CCL2*, *IFNB1*, *IL1A*, *IFIT3*
Pathogen-induced cytokine storm signaling pathway	−5.091	4.85 × 10^−23^	50	*IL33*, *CCL2*, *CXCL9*, *CSF2*, *NOS2*
Role of pattern recognition receptors in recognition of bacteria and viruses	−2.887	1.10 × 10^−17^	29	*IL33*, *CSF2*, *TNFSF10*, *IFNB1*, *IL1A*
Multiple sclerosis signaling pathway	−3.402	1.03 × 10^−12^	28	*IL33*, *CSF2*, *TNFSF10*, *IL1A*, *IL36A*
NOD1/2 signaling pathway	−3.138	8.86 × 10^−13^	26	*IL33*, *CCL2*, *CSF2*, *TNFSF10*, *NOS2*
TREM1 signaling	−3.638	3.42 × 10^−12^	17	*CCL2*, *CSF2*, *CD86*, *IL6*, *IL1B*
Macrophage classical activation signaling pathway	−3.400	5.92 × 10^−12^	25	*IL33*, *CSF2*, *TNFSF10*, *NOS2*
IL-17 signaling	−4.146	6.02 × 10^−09^	21	*IL33*, *CCL2*, *MMP3*, *CSF2*, *NOS2*
Interferon signaling	−3.162	9.25 × 10^−09^	10	IFIT1 *, *IFNB1*, IFIT3 *, *ISG15*
Neuroinflammation signaling pathway	−2.449	1.84 × 10^−08^	27	*CCL2*, *MMP3*, *NOX1*, *NOS2*
Differential regulation of cytokine production in macrophages and T-helper cells	−2.646	1.19 × 10^−07^	7	*CCL2*, *CSF2*, *IL6*, *IL1B*, *CCL4*
LXR/RXR activation	2.840	3.04 × 10^−07^	15	*IL33*, *CCL2*, *NOS2*, *IL1A*, *IL36A*
Toll-like receptor signaling	−2.111	3.68 × 10^−07^	12	*IL33*, *IL1A*, *IL36A*, *IL1B*, *TLR8*
LPS-IL-1-mediated inhibition of RXR function	−2.000	1.81 × 10^−05^	19	*IL33*, *IL1A*, *IL36A*, *IL1B*, *FABP4*
Glutathione-mediated detoxification *	1.633	2.58 × 10^−05^	7	*Gsta4*, GSTA5 *, *GSTA3*, *MGST2*
HMGB1 signaling	−2.646	5.89 × 10^−05^	14	*IL33*, *CCL2*, *CSF2*, *TNFSF10*, *IL1A*
P38 MAPK signaling	−2.530	1.64 × 10^−04^	11	*IL33*, *IL1A*, *IL36A*, *IL1B*, *PLA2G4C*
PPAR signaling	1.265	2.75 × 10^−05^	10	*IL33*, *IL1A*, *IL36A*, *IL1B*
NRF2-mediated oxidative stress response *	1.414	2.22 × 10^−03^	14	*GSTA5*, *GSTA3*, *NQO1*, *PRDX1*

* The genes involved in these pathways were upregulated.

## Data Availability

Data presented in this study are available within this article and Supplementary Materials.

## Supplementary Materials

The following supporting information can be downloaded at: https://www.mdpi.com/article/10.3390/md21110553/s1, ^1^H NMR, HSQC, HSQC-TOCSY, HMBC, 1D TOCSY for 7(*E*)-9-keto-hexadec-7-enoic acid (**1**) in CDCl_3_; ^1^H NMR, HSQC, COSY, HMBC, TOCSY for 7(*E*)-9*S*-hydroxy-hexadec-7-enoic acid (**2**) in CDCl_3_; ^1^H NMR, HSQC, band-selective HSQC for 9(*Z*)-hexadec-9-enoic acid (**3**) in CDCl_3_; ^1^H NMR spectrum for hexadecanoic acid (**4**) in CDCl_3_; Table S1: NMR spectral data for 7(*E*)-9*S*-hydroxy-hexadec-7-enoic acid (**2**) and 9(*Z*)-hexadec-9-enoic acid (**3**); Figure S1: ESI-MS/MS spectra for **1**; Figures S2 and S3: HRMS data for (**1**−**4**); Figure S4: LRMS data for ozonolysis products of **2** and **3**; Figure S5: homonuclear decoupling of **1** and *E*/*Z* commercially available standards; Figure S6: upstream regulator network for LPS; Figure S7: regulator effect networks of the Nrf2 activators.

## Sample Availability

Data are included in the article or Supporting Information. Raw data will be made available upon request.
