# Supplementary material for: Discovery and Anti-Inflammatory Activity of a Cyanobacterial Fatty Acid Targeting the Keap1/Nrf2 Pathway
Source: Mar Drugs. 2023 Oct 25;21(11):553. doi: 10.3390/md21110553 (PMC10672429; doi:10.3390/md21110553)
Supplement: Supplementary file 1 [file marinedrugs-21-00553-s001.zip › marinedrugs-2639355-supplementary.pdf]

Supporting Information

Discovery and Anti-inflammatory Activity of a Cyanobacterial Fatty Acid Targeting the Keap1/Nrf2 Pathway

Fatma H. Al-Awadhi[1,2], Emily F. Simon[1], Na Liu[1,3], Ranjala Ratnayake[1], Valerie J. Paul[4], and Hendrik Luesch[1,*]

[1]*Department of Medicinal Chemistry and Center for Natural Products, Drug Discovery and Development (CNPD3), University of Florida, Gainesville; fatalawadhi@ufl.edu (F.H.A.); simonemily@ufl.edu (E.F.S.); rratnayake@cop.ufl.edu (R.R.); luesch@cop.ufl.edu (H.L.)*
[2]*Department of Pharmaceutical Chemistry, Faculty of Pharmacy, Kuwait University, Kuwait; fatma.h@ku.edu.kw (F.H.A.)*
[3]*University of Jinan, School of Biological Science and Technology, Jinan 250022, China; mls_liun@ujn.edu.cn* (N.L.)
[4]*Smithsonian Marine Station, Fort Pierce, Florida, USA; paul@si.edu (V.J.P.)*

***** Correspondence: luesch@cop.ufl.edu

Contents **Page Number**

^{1}H NMR Spectrum of 7(*E*)-9-keto-hexadec-7-enoic acid (**1**) in $CDCl_3$ (800 MHz) S3

HSQC Spectrum of 7(*E*)-9-keto-hexadec-7-enoic acid (**1**) in $CDCl_3$ (800 MHz) S4

Selective 1D TOCSY Spectra of 7(*E*)-9-keto-hexadec-7-enoic acid (**1**) in $CDCl_3$ (800 MHz) S5

HSQC-TOCSY Spectrum of 7(*E*)-9-keto-hexadec-7-enoic acid (**1**) in $CDCl_3$ (800 MHz) S6

HMBC Spectrum of 7(*E*)-9-keto-hexadec-7-enoic acid (**1**) in $CDCl_3$ (600 MHz) S7

^{1}H NMR Spectrum of 7(*E*)-9*S*-hydroxy-hexadec-7-enoic acid (**2**) in $CDCl_3$ (600 MHz) S8

HSQC Spectrum of 7(*E*)-9*S*-hydroxy-hexadec-7-enoic acid (**2**) in $CDCl_3$ (600 MHz) S9

COSY Spectrum of 7(*E*)-9*S*-hydroxy-hexadec-7-enoic acid (**2**) in $CDCl_3$ (600 MHz) S10

HMBC Spectrum of 7(*E*)-9*S*-hydroxy-hexadec-7-enoic acid (**2**) in $CDCl_3$ (600 MHz) S11

TOCSY Spectrum of 7(*E*)-9*S*-hydroxy-hexadec-7-enoic acid (**2**) in $CDCl_3$ (600 MHz) S12

^{1}H NMR Spectrum of 9(*Z*)-hexadec-9-enoic acid (**3**) in $CDCl_3$ (600 MHz) S13

HSCQ Spectrum of 9(*Z*)-hexadec-9-enoic acid (**3**) in $CDCl_3$ (600 MHz) S14

Band-Selective HSCQ Spectrum of 9(*Z*)-hexadec-9-enoic acid (**3**) in $CDCl_3$ (600 MHz) S15

^{1}H NMR Spectrum of hexadecanoic acid (**4**) in $CDCl_3$ (600 MHz) S16

^{1}H NMR Spectrum of 7(*E*)-9-keto-hexadec-7-enoic acid (**1**) from DRTO-73 in $CDCl_3$ (600 MHz) S17

HSQC Spectrum of 7(*E*)-9-keto-hexadec-7-enoic acid (**1**) from DRTO-73 in $CDCl_3$ (600 MHz) S18

HSQC-TOCSY Spectrum of 7(*E*)-9-keto-hexadec-7-enoic acid (**1**) from DRTO-73 in $CDCl_3$ (600 MHz) S19

Selective 1D TOCSY Spectrum of 7(*E*)-9-keto-hexadec-7-enoic acid (**1**) from DRTO-73 in $CDCl_3$ (600 MHz) S20

Table S1: NMR spectral data for compounds (**2**) and (**3**) S21

Figure S1: LRMS/MS spectra of 7(*E*)-9-keto-hexadec-7-enoic acid (**1**) in the negative mode S22

Figure S2: HRMS spectra of 7(*E*)-9-keto-hexadec-7-enoic acid (**1**) and (**2**) 7(*E*)-9*S*-hydroxy-hexadec-7-enoic acid S23

Figure S3: HRMS spectra of 9(*Z*)-hexadec-9-enoic acid (**3**) and hexadecanoic acid (**4**) S24

Figure S4: LRMS spectra of the ozonolysis products of (**2**) and (**3**) S25

Figure S5: Homo-nuclear decoupling of **1** and commercially available *E*/*Z* standards in $CDCl_3$ (600 MHz) S26

Figure S6: Upstream regulator network of LPS S27

Figure S7: Regulator effect networks of the Nrf2 activators fingolimod, resveratrol, and curcumin S28

^{1}H NMR Spectrum of 7(*E*)-9-keto-hexadec-7-enoic acid (**1**) in $CDCl_3$ (800 MHz)

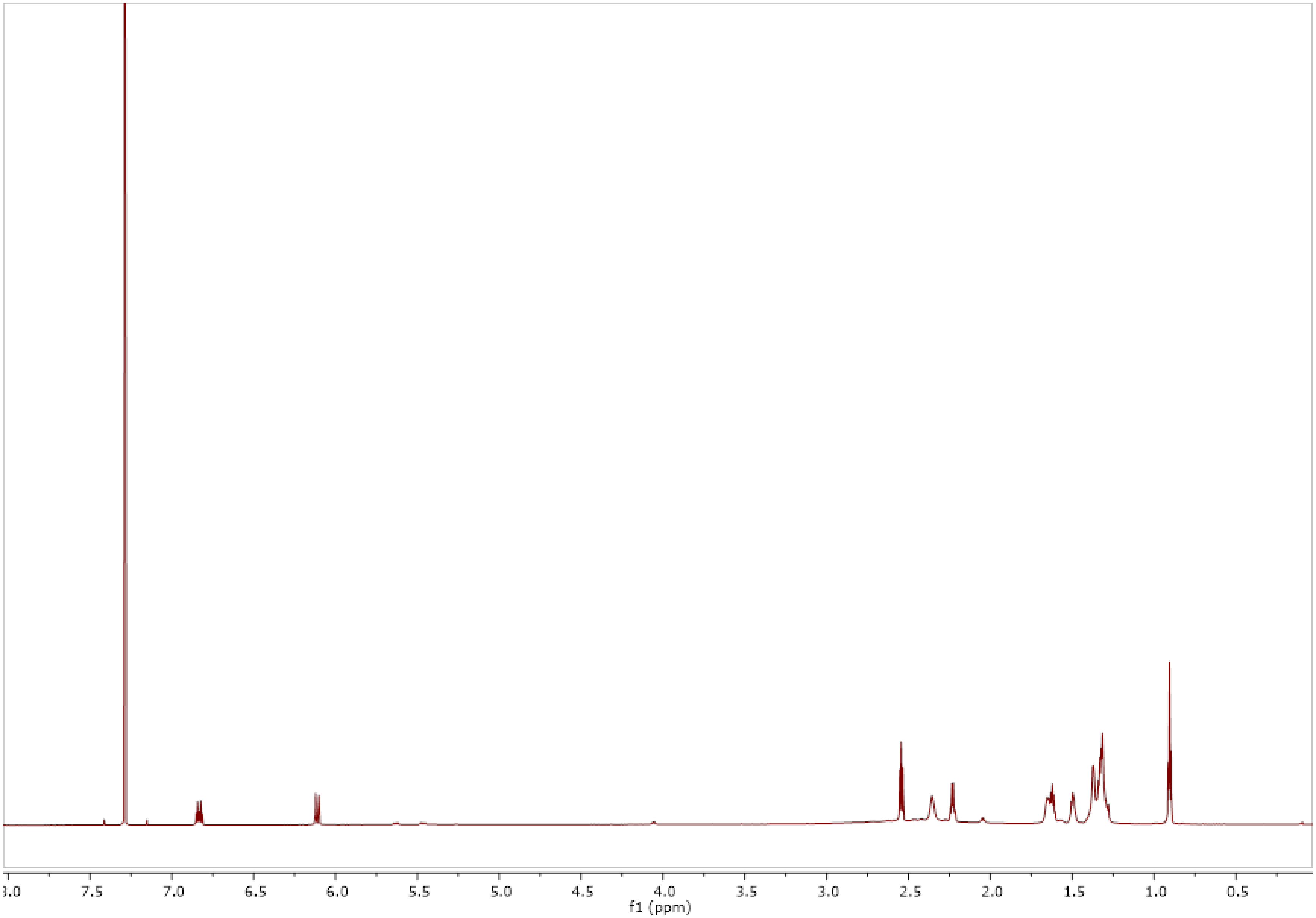

HSQC Spectrum of 7(*E*)-9-keto-hexadec-7-enoic acid (**1**) in $CDCl_3$ (800 MHz)

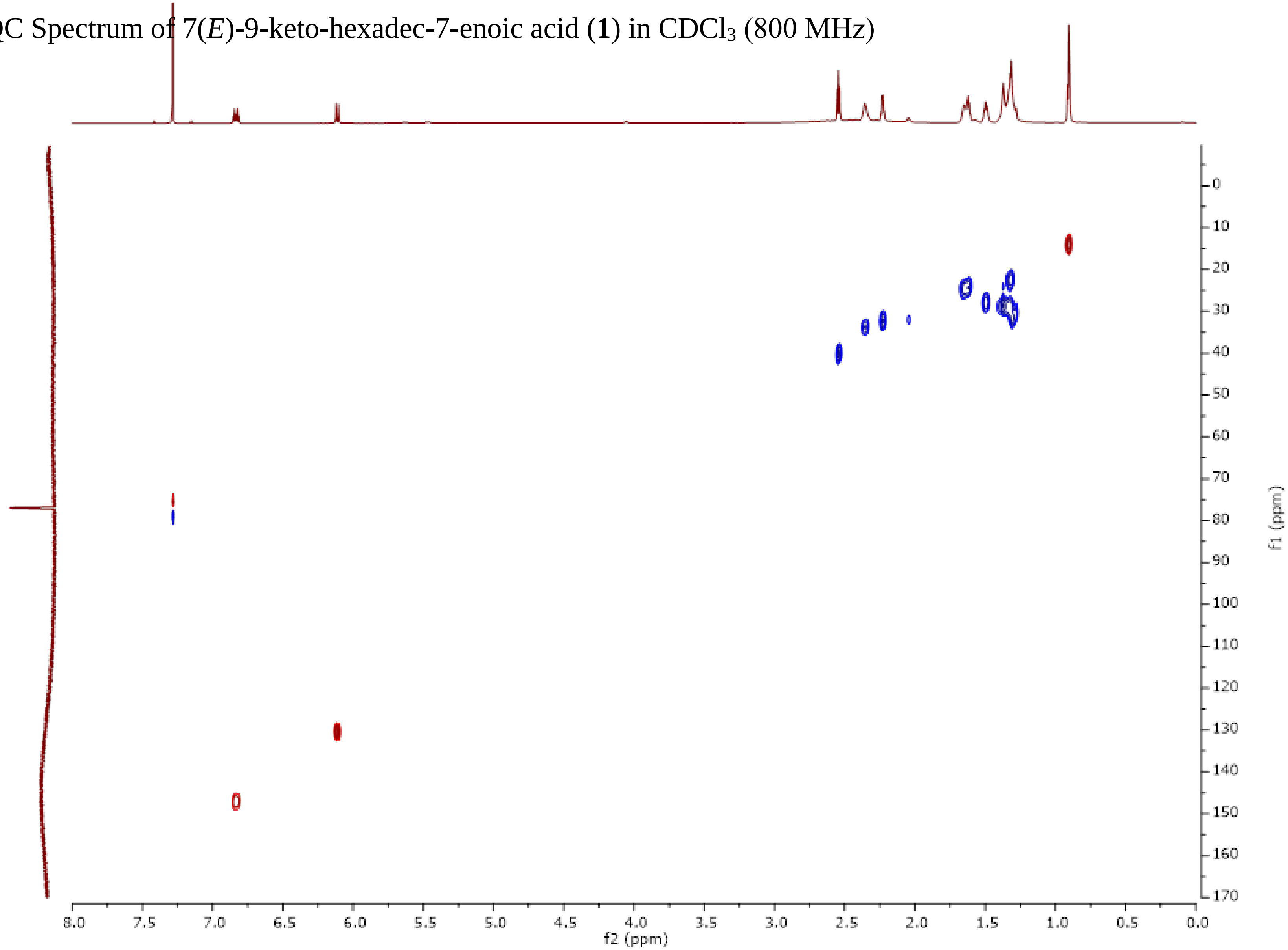

Selective 1D TOCSY Spectra of 7(*E*)-9-keto-hexadec-7-enoic acid (**1**) in $CDCl_3$ (800 MHz)

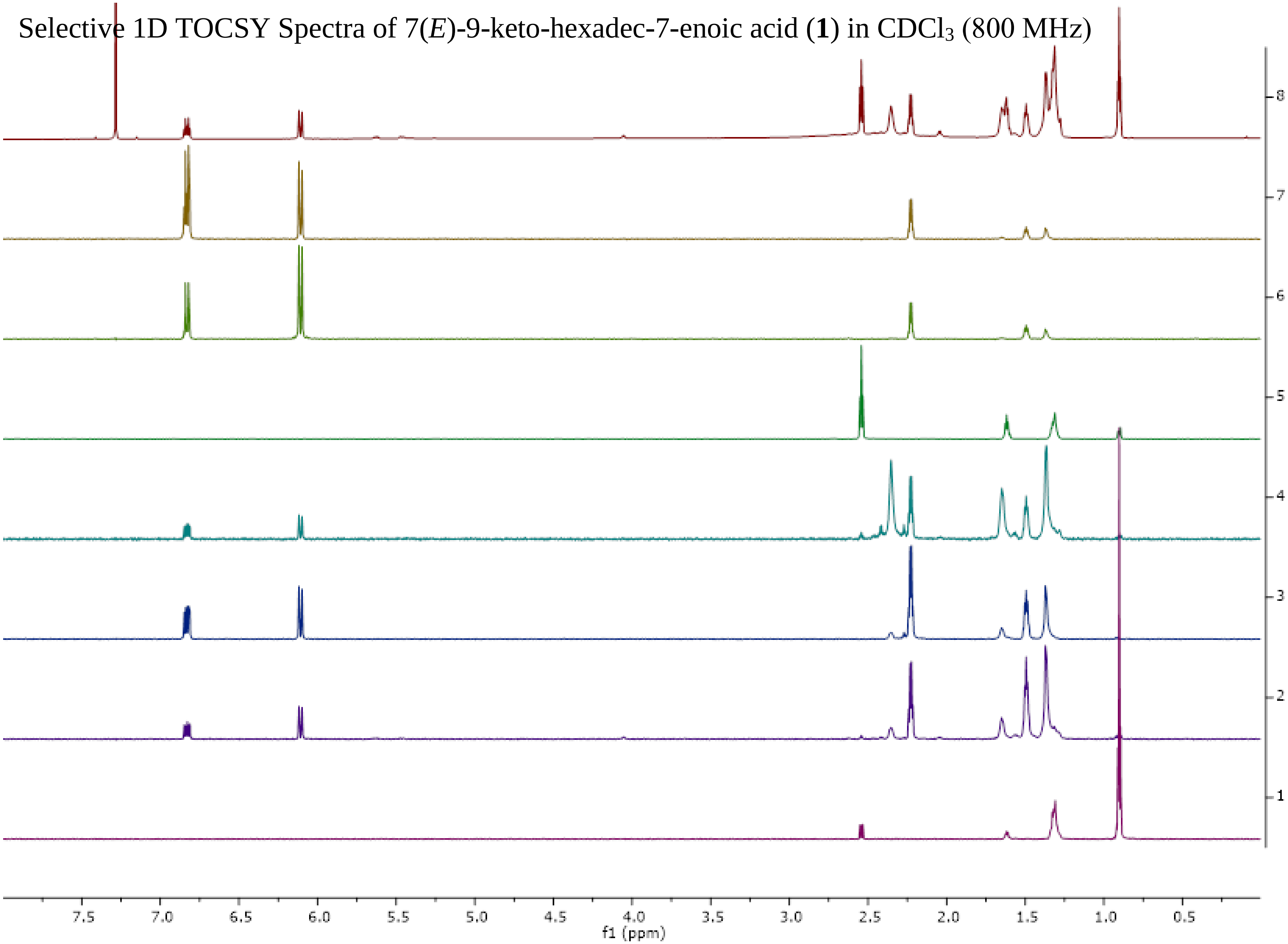

HSQC-TOCSY Spectrum of 7(*E*)-9-keto-hexadec-7-enoic acid (**1**) in $CDCl_3$ (800 MHz)

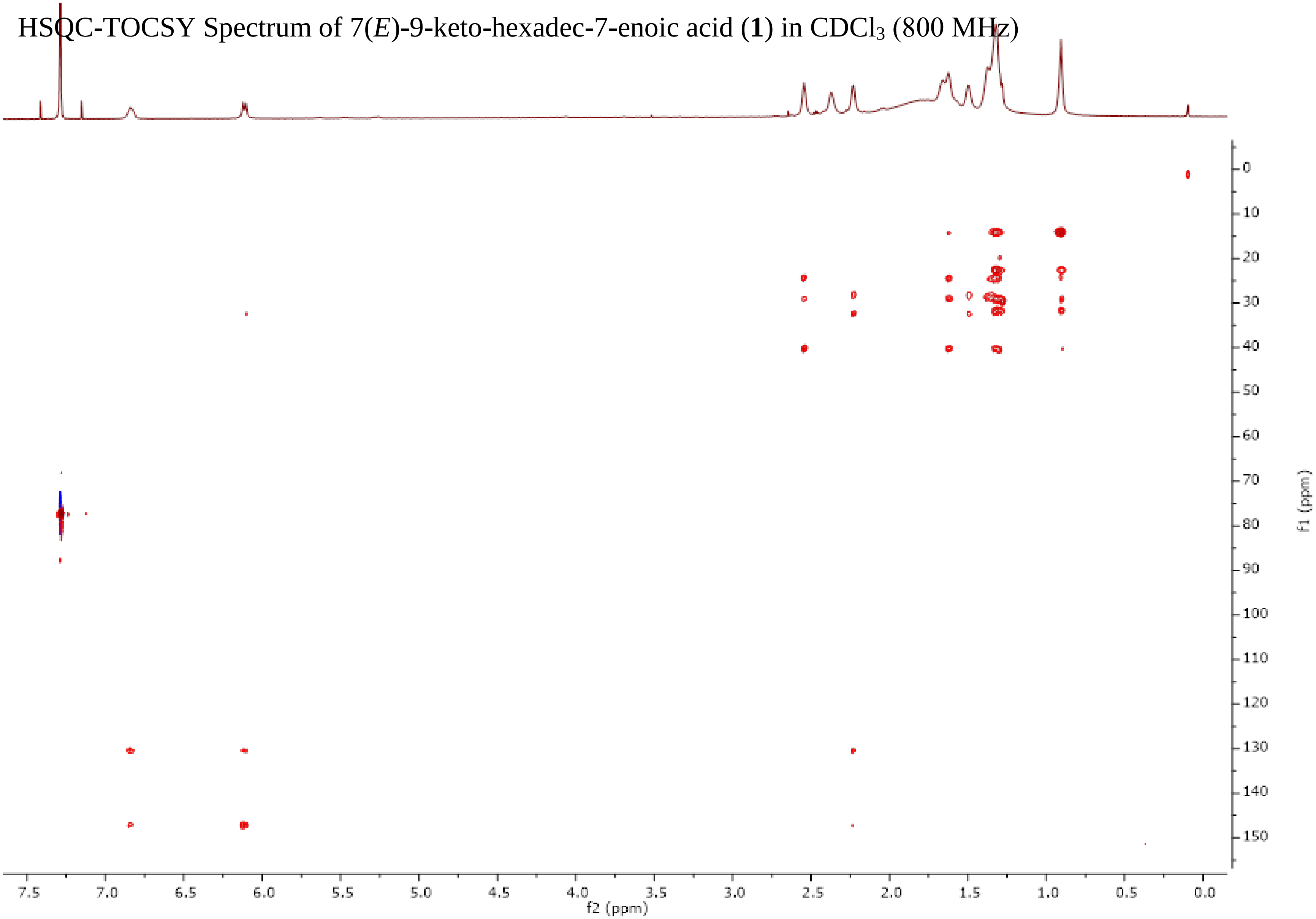

HMBC Spectrum of 7(*E*)-9-keto-hexadec-7-enoic acid (**1**) in $CDCl_3$ (600 MHz)

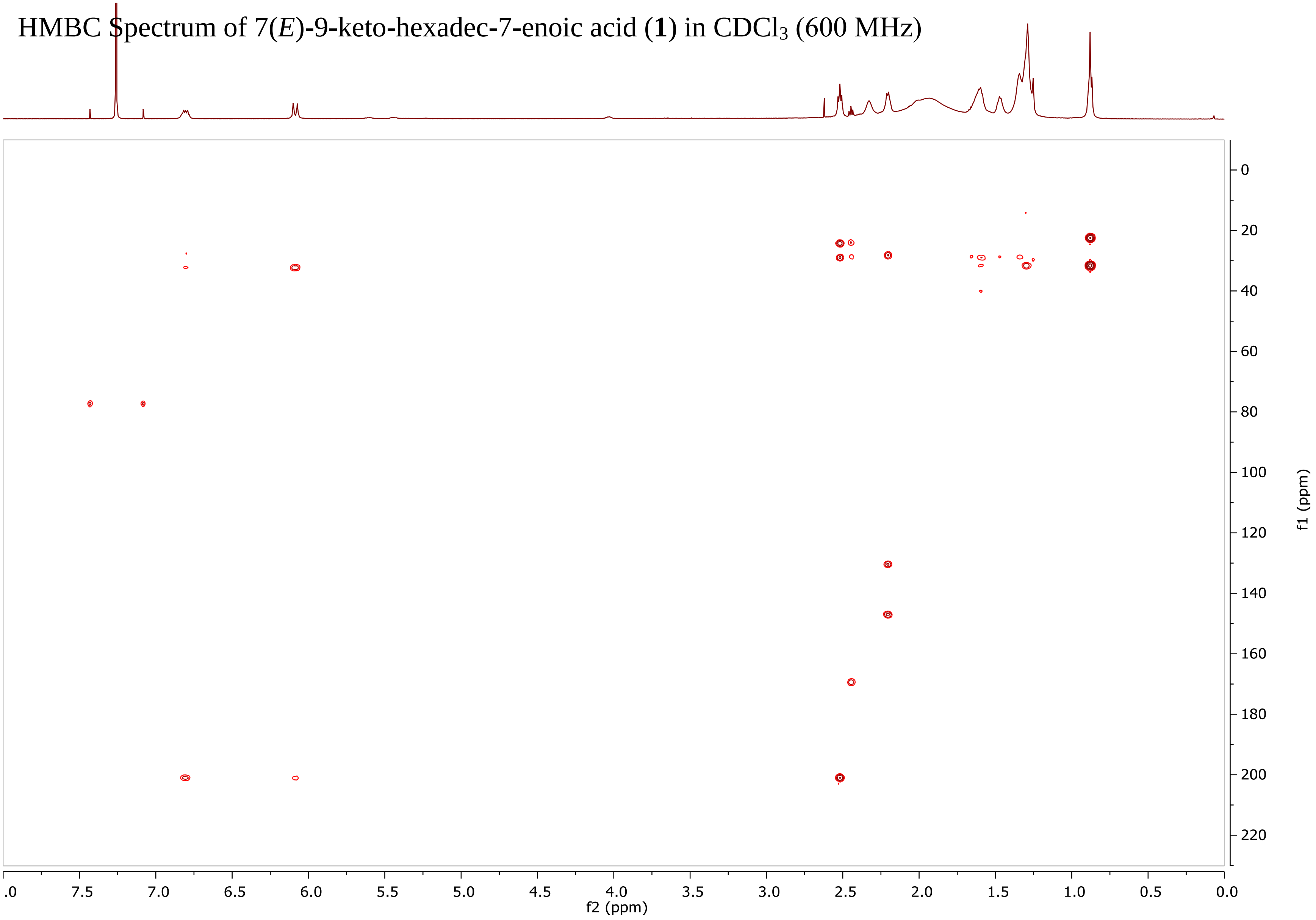

^{1}H NMR Spectrum of 7(*E*)-9*S*-hydroxy-hexadec-7-enoic acid (**2**) in $CDCl_3$ (600 MHz)

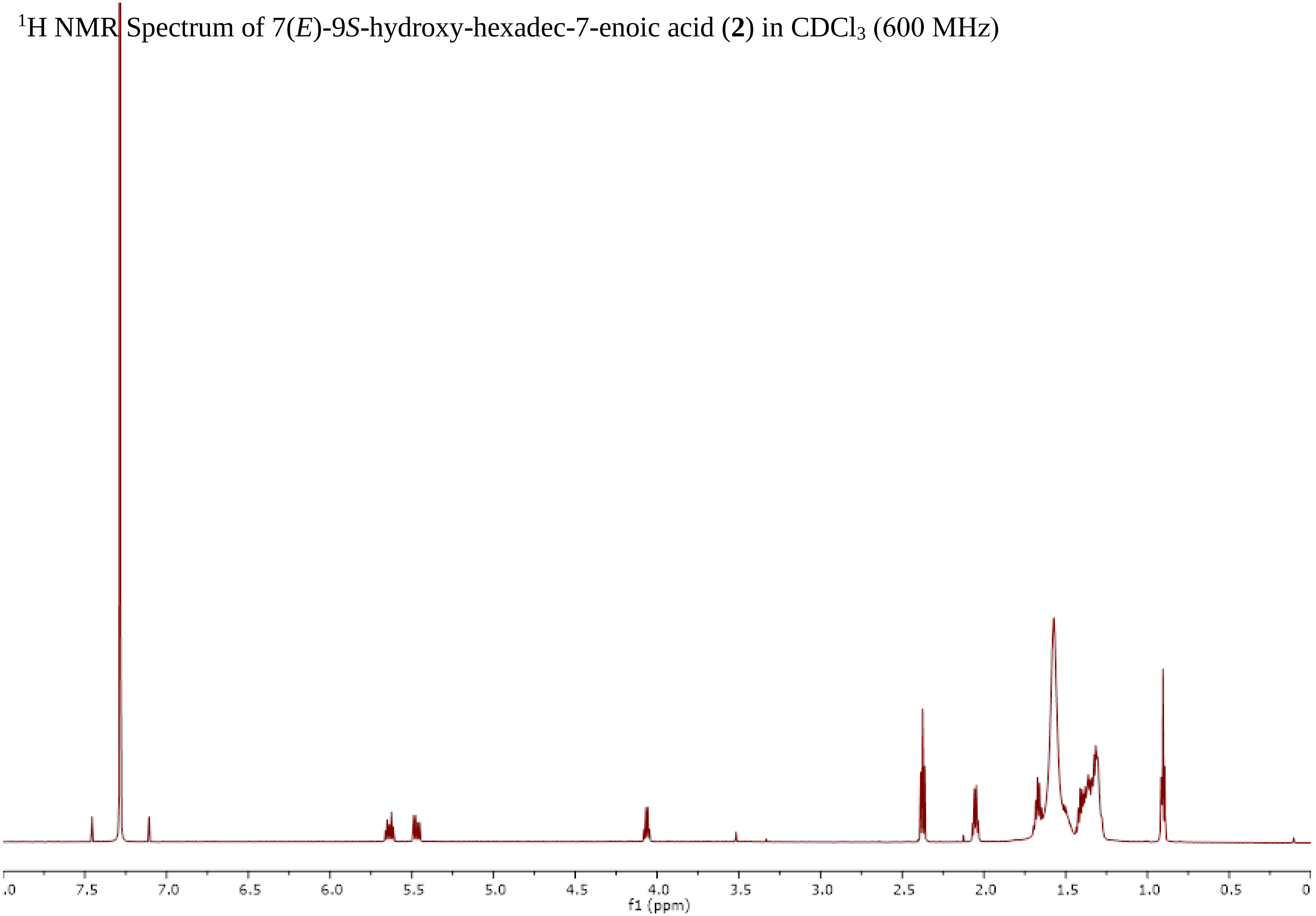

HSQC Spectrum of 7(*E*)-9*S*-hydroxy-hexadec-7-enoic acid (**2**) in $CDCl_3$ (600 MHz)

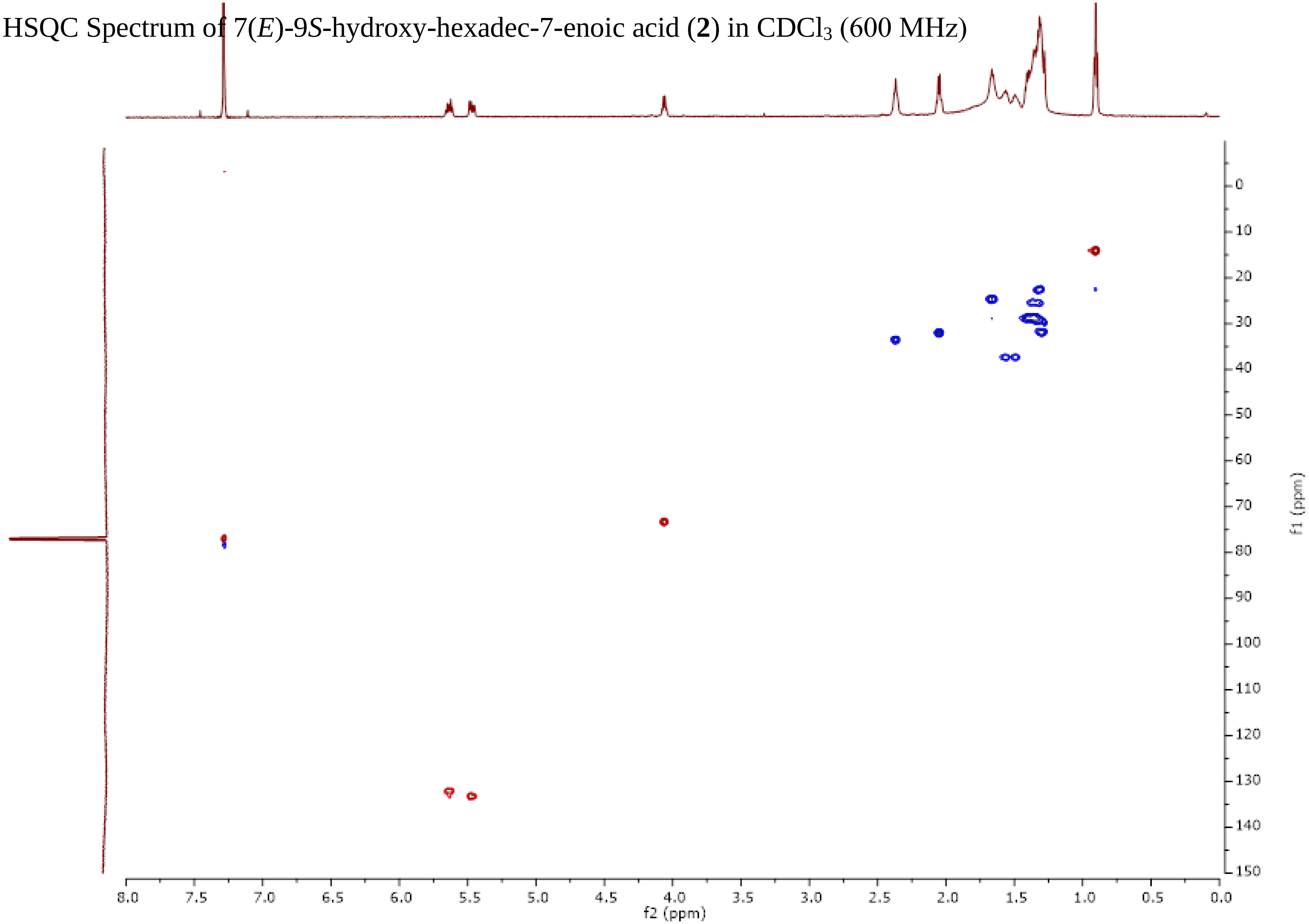

COSY Spectrum of 7(*E*)-9*S*-hydroxy-hexadec-7-enoic acid (**2**) in $CDCl_3$ (600 MHz)

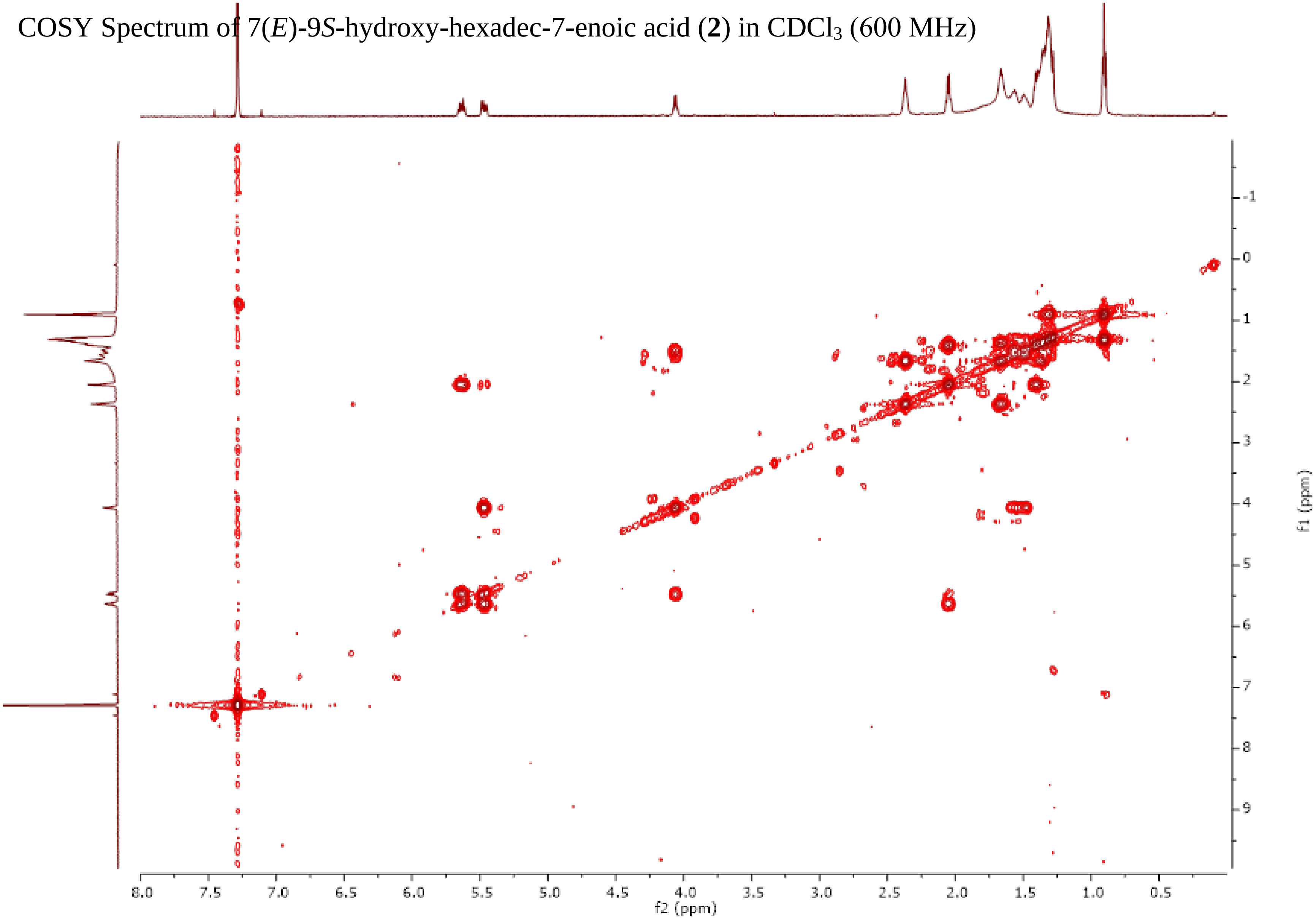

HMBC Spectrum of 7(*E*)-9*S*-hydroxy-hexadec-7-enoic acid (**2**) in $CDCl_3$ (600 MHz)

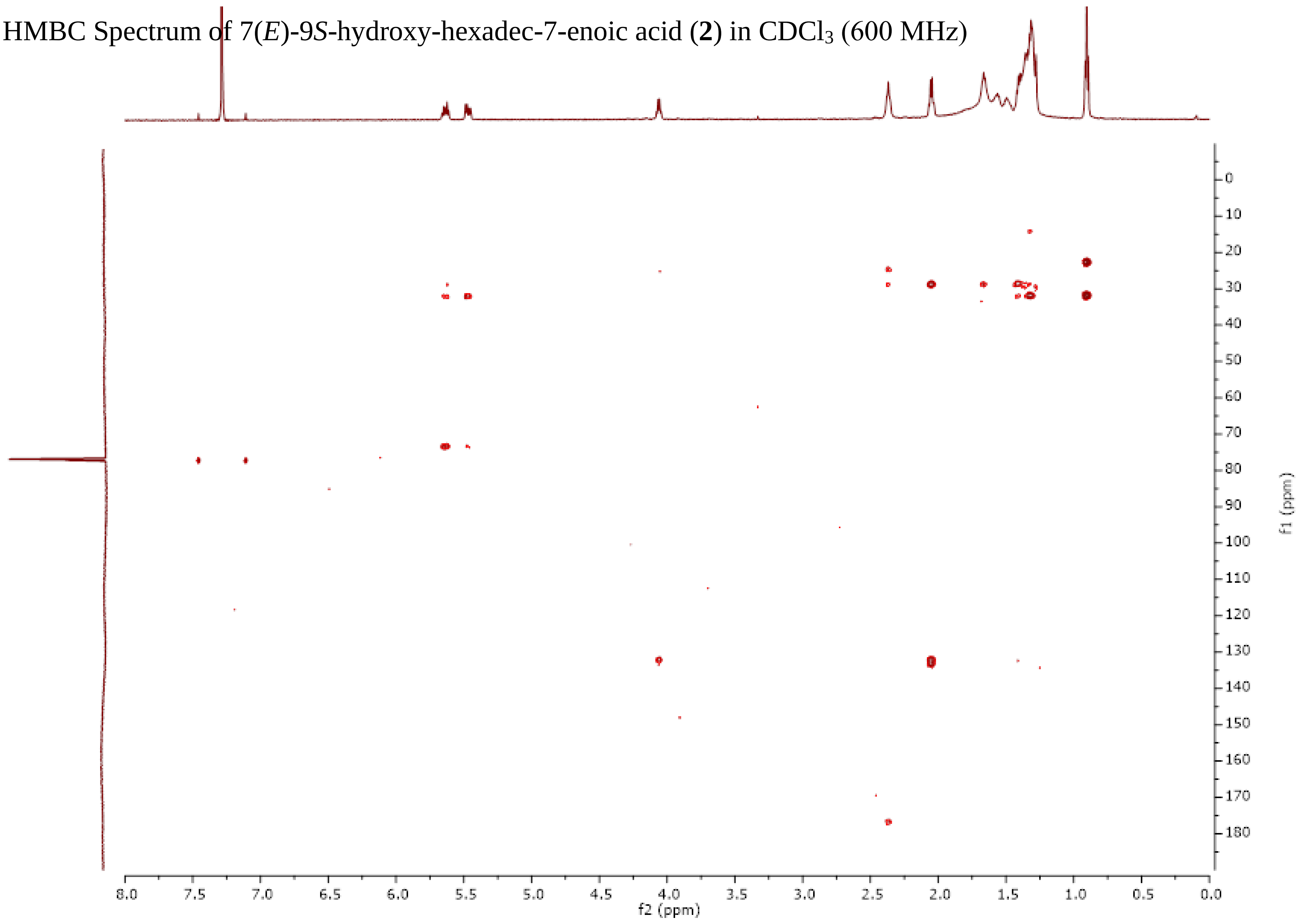

TOCSY Spectrum of 7(*E*)-9*S*-hydroxy-hexadec-7-enoic acid (**2**) in $CDCl_3$ (600 MHz)

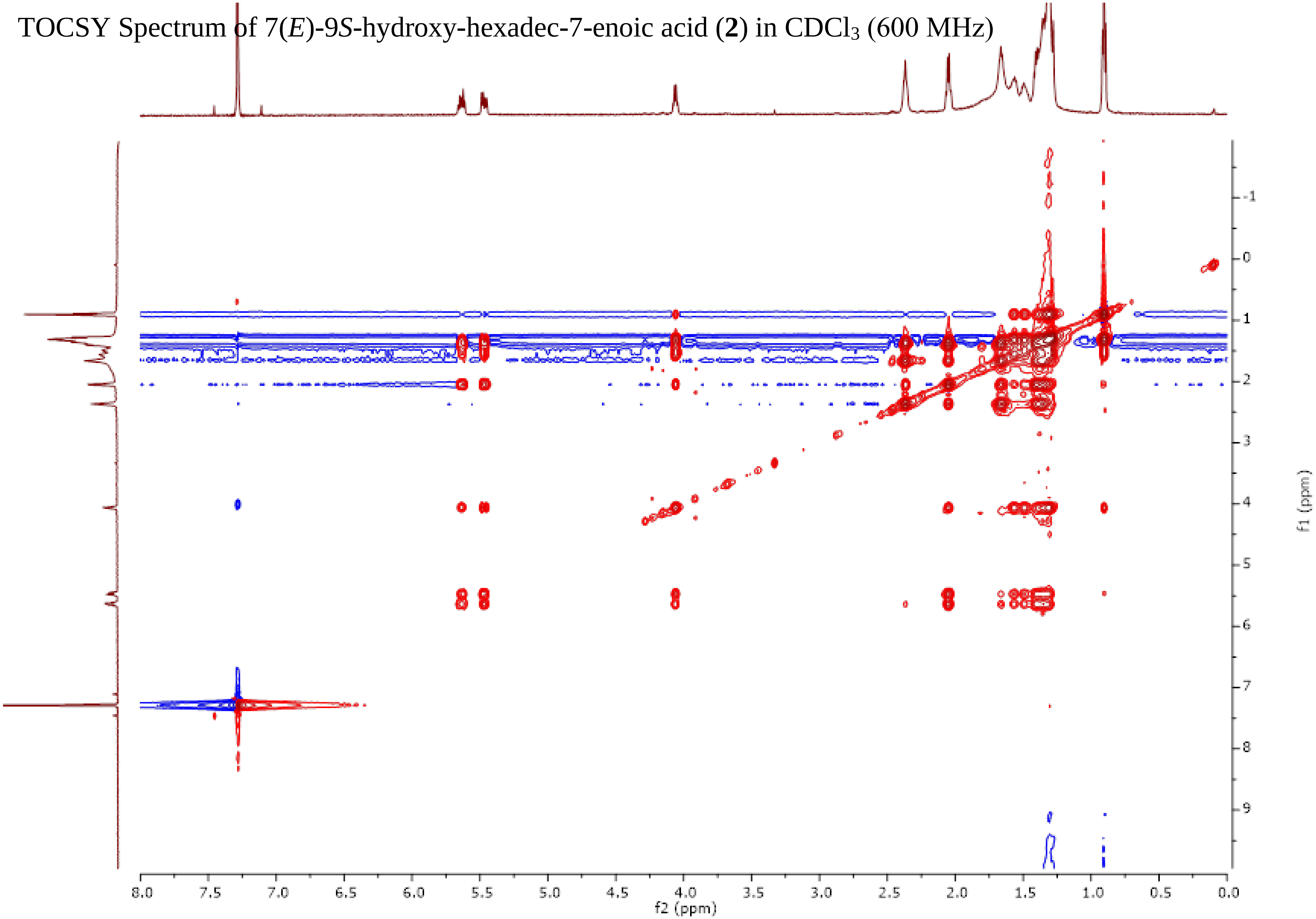

^{1}H NMR Spectrum of 9(*Z*)-hexadec-9-enoic acid (**3**) in $CDCl_3$ (600 MHz)

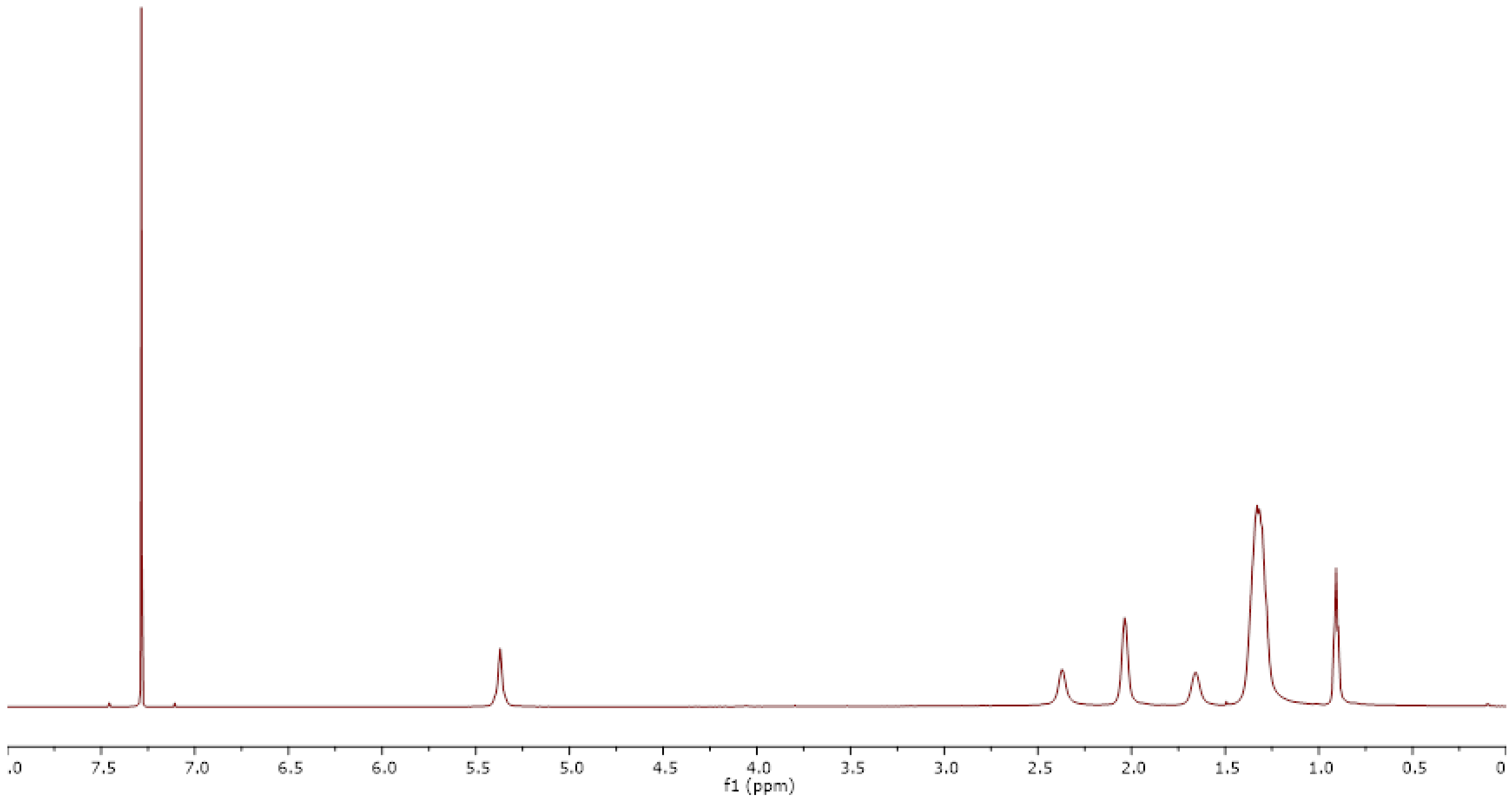

HSQC Spectrum of 9(*Z*)-hexadec-9-enoic acid (**3**) in $CDCl_3$ (600 MHz)

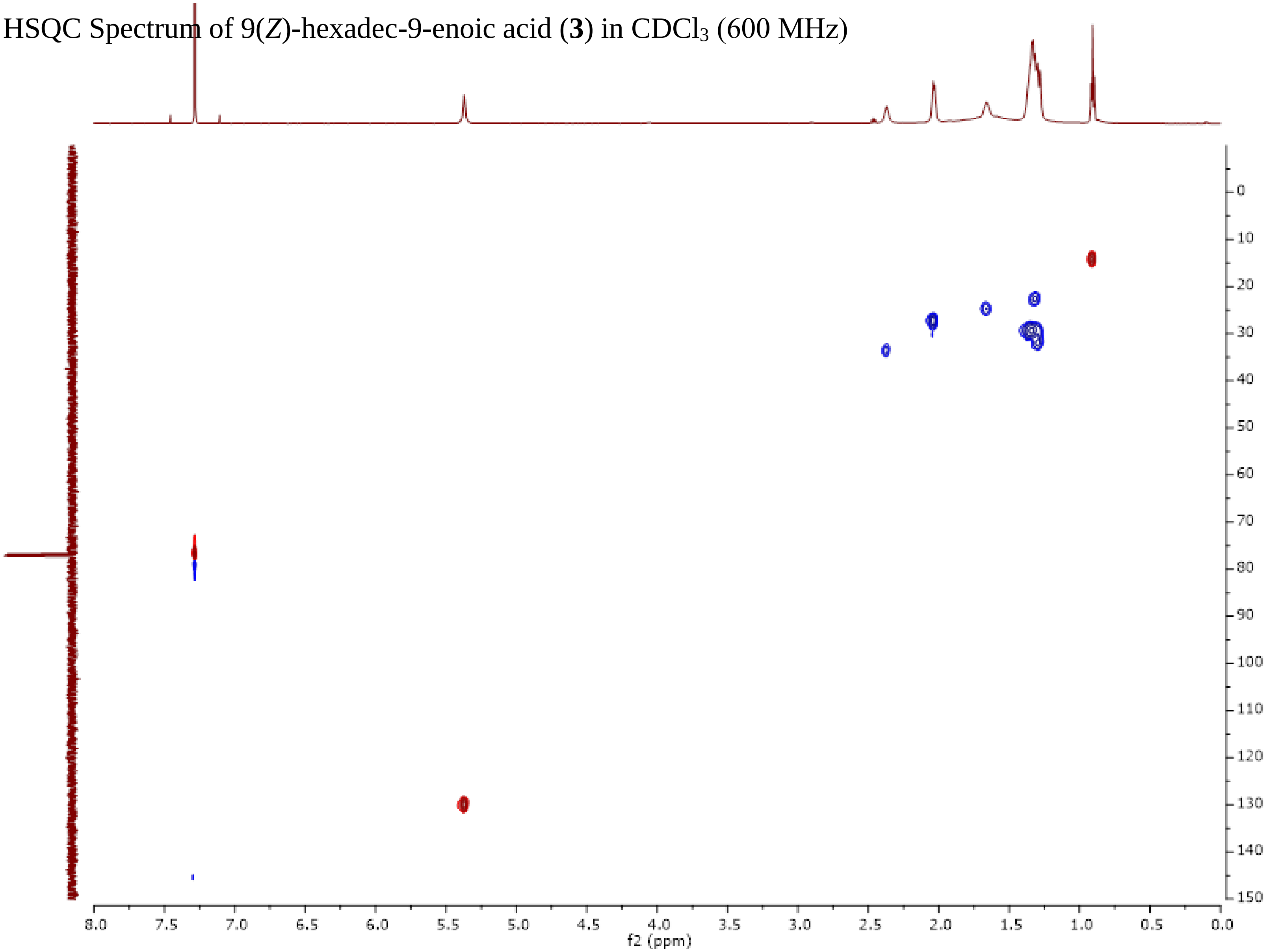

Band-Selective HSQC Spectrum of 9(*Z*)-hexadec-9-enoic acid (**3**) in $CDCl_3$ (600 MHz)

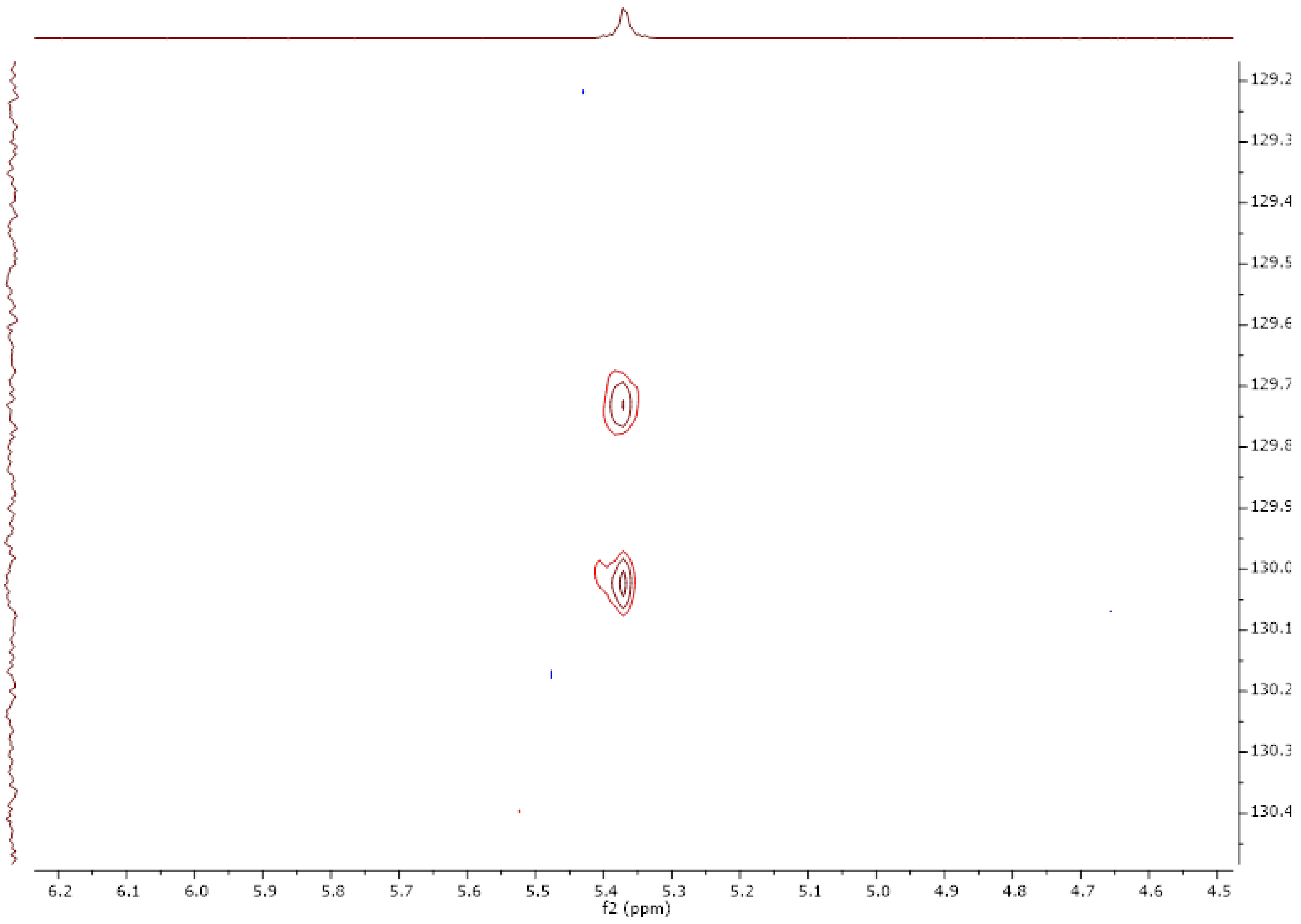

^{1}H NMR Spectrum of hexadecanoic acid (**4**) in $CDCl_3$ (600 MHz)

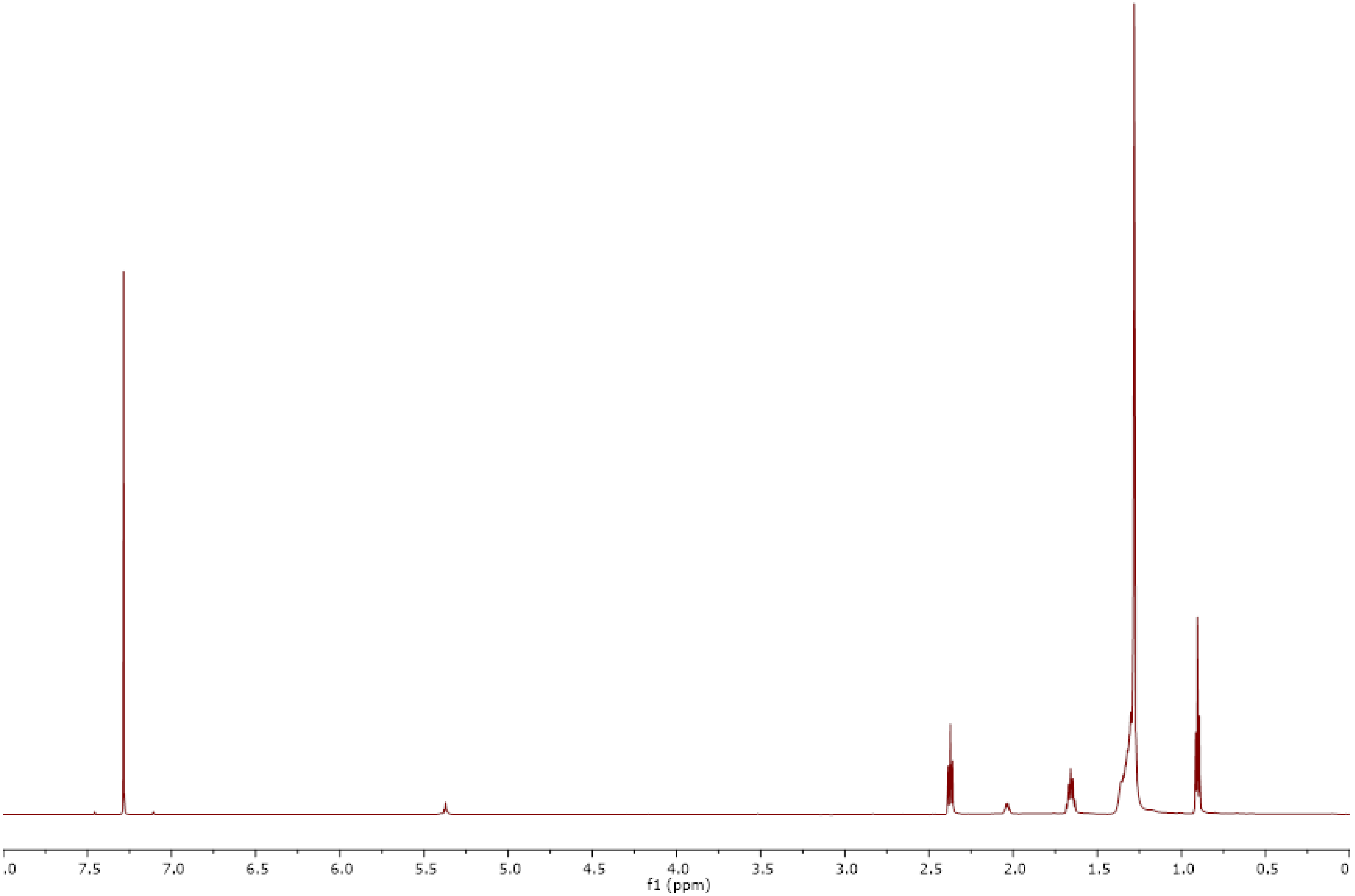

^{1}H NMR Spectrum of 7(*E*)-9-keto-hexadec-7-enoic acid (**1**) from DRTO-73 (Loggerhead Key) in $CDCl_3$ (600 MHz)

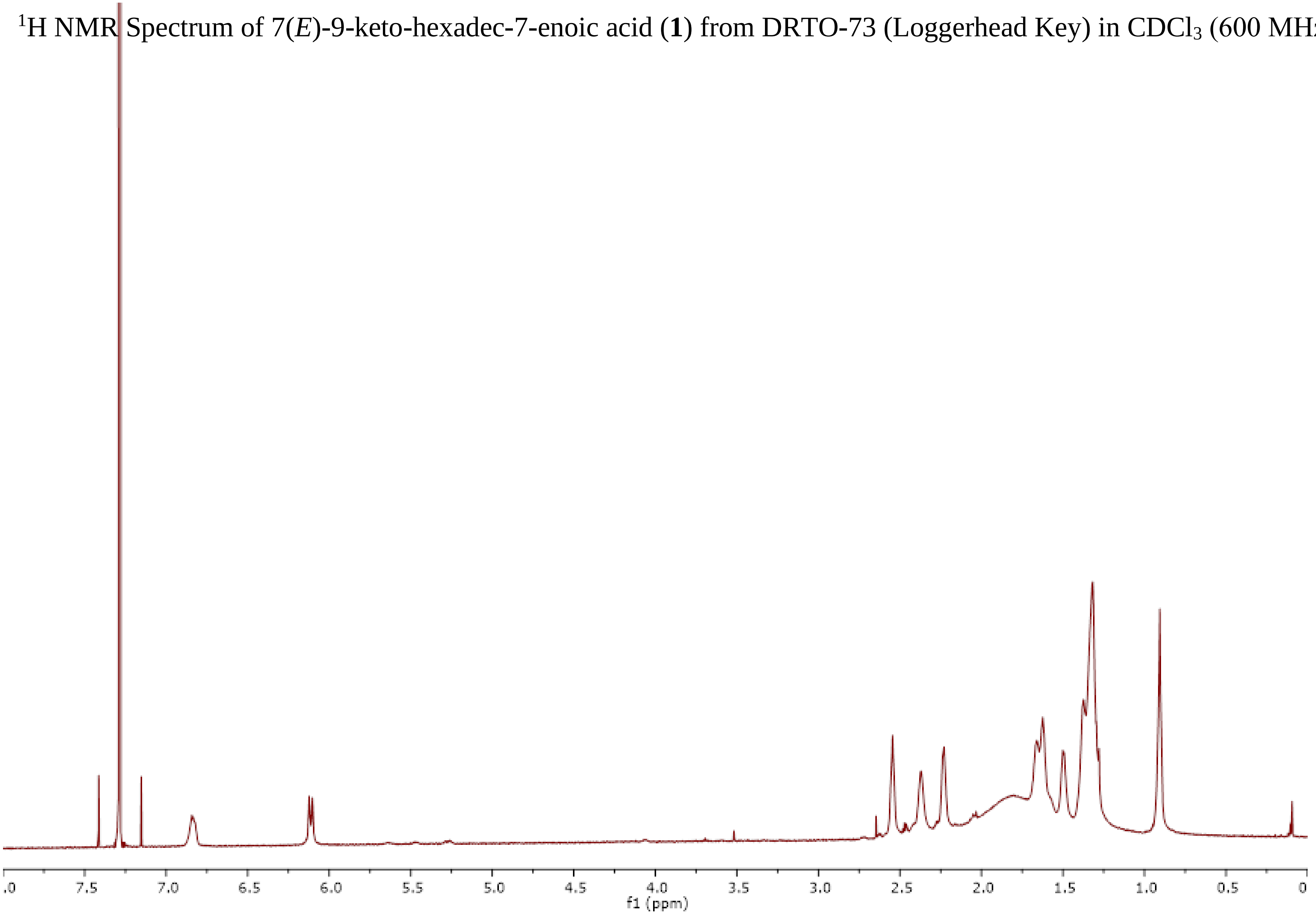

HSQC Spectrum of 7(*E*)-9-keto-hexadec-7-enoic acid (**1**) from DRTO-73 (Loggerhead Key) in $CDCl_3$ (600 MHz)

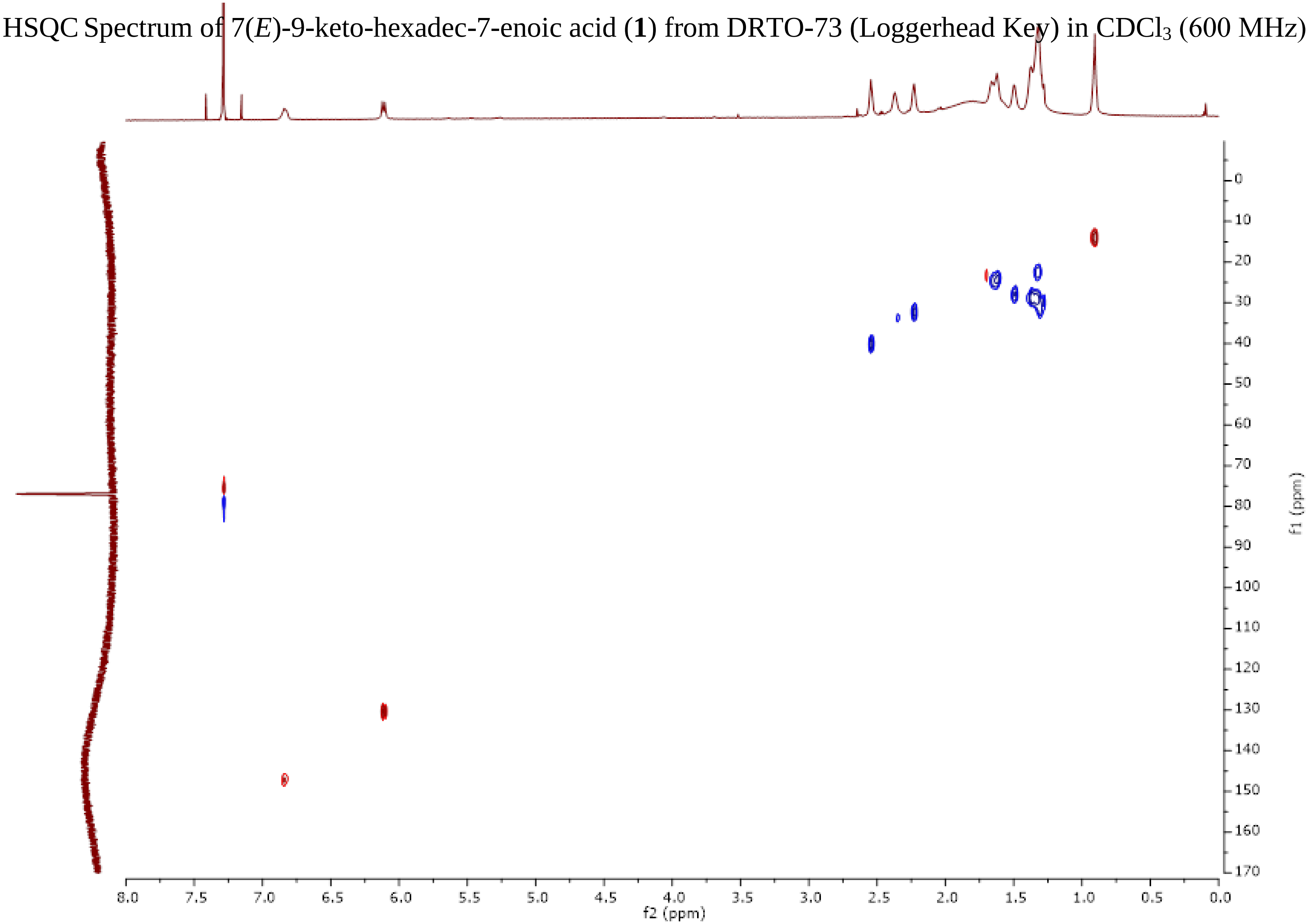

HSQC-TOCSY Spectrum of 7(*E*)-9-keto-hexadec-7-enoic acid (**1**) from DRTO-73 (Loggerhead Key) in $CDCl_3$ (600 MHz)

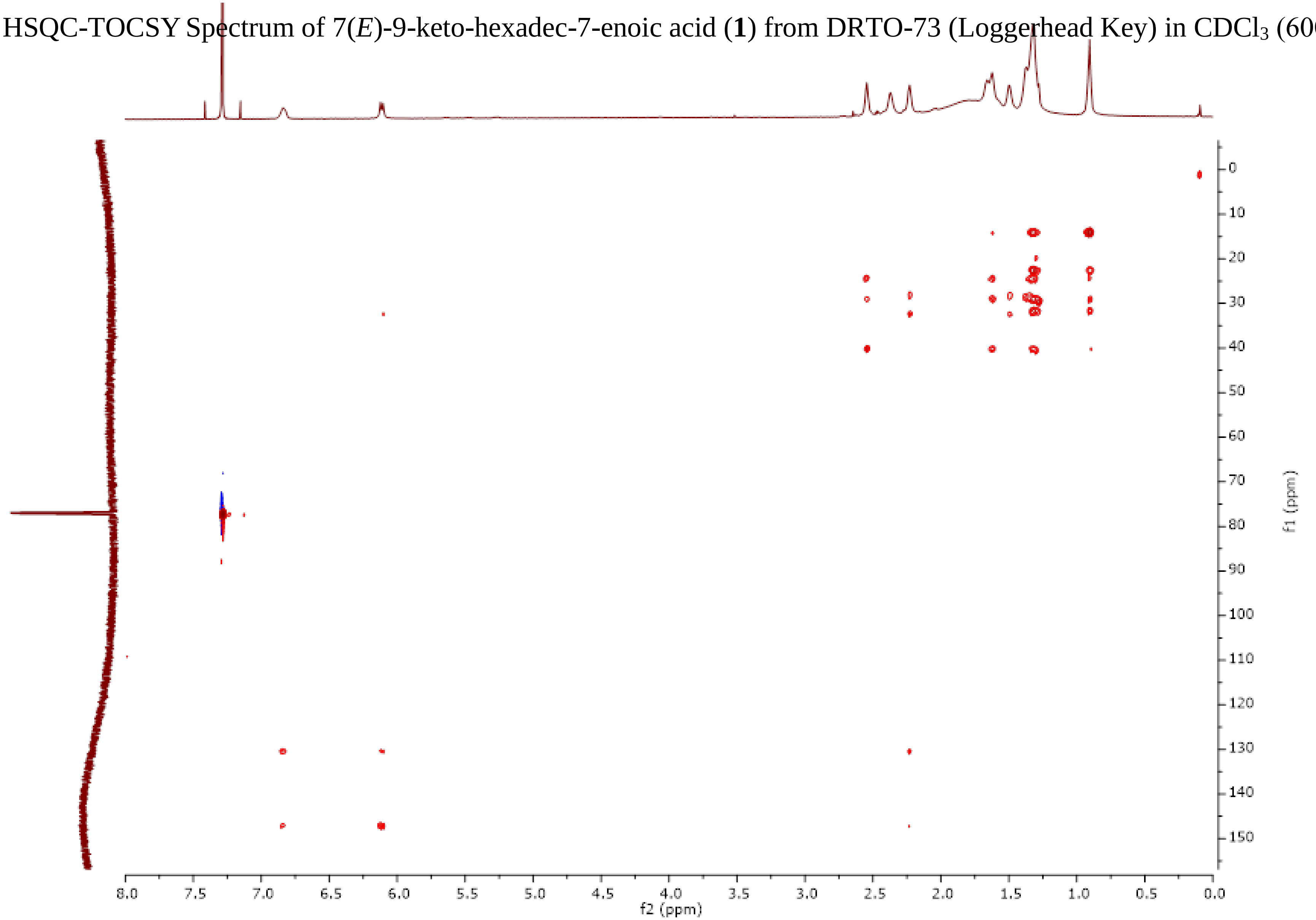

Selective 1D TOCSY Spectrum of 7(*E*)-9-keto-hexadec-7-enoic acid (**1**) from DRTO-73 (Loggerhead Key) in $CDCl_3$ (600 MHz)

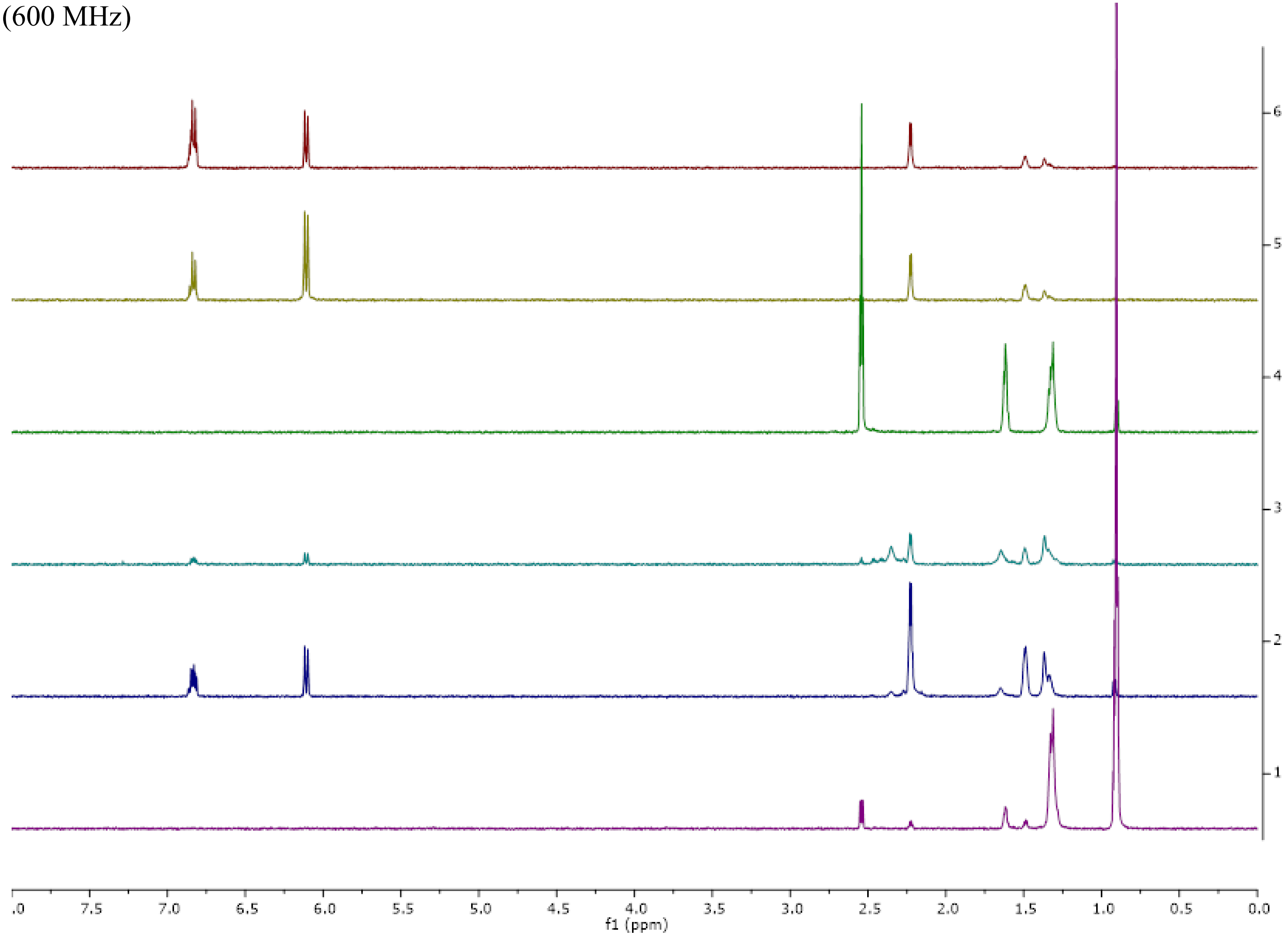

Table S1: NMR spectral data for 7(*E*)-9*S*-hydroxy-hexadec-7-enoic acid (**2**) and hexadec-9-enoic acid (**3**) in $CDCl_3$ (600 MHz).

C/H No.	**2**				**3**	
	**δ_H (*J* in Hz)**	**δ_C[a], mult[b]**	**COSY**	**HMBC**	**δ_H (*J* in Hz)**	**δ_C[a], mult[b]**
OH	–	–			–	–
1	–	176.6, qC			–	qC
2	2.37, t (7.4)	33.5, CH_2	H_2-3	1, 3, 4	2.37, t (7.4)	33.2, CH_2
3	1.67, m	24.5, CH_2	H_2-2, H_2-4	4, 5	1.67, m	24.8, CH_2
4	1.37, m	28.7, CH_2			1.32, m	29.4, CH_2
5	1.40, m	28.7, CH_2	H_2-6	3, 6	1.32, m	29.4, CH_2
6	2.05, dt (6.9, 7.1)	32.1, CH_2	H_2-5, H-7	4, 5, 7, 8	2.02, dt (6.9, 6.3)	29.4, CH_2
7	5.63, dt (15.3, 6.8)	132.1, CH	H_2-6, H-8	6, 9	5.34, dt (11.0, 5.5)	29.4, CH_2
8	5.47, dd (15.3, 7.1)	133.2, CH	H-9	6, 9	5.34, dt (11.0, 5.5)	27.4, CH_2
9	4.06, dt (7.1, 6.8)	73.3, CH	H-8, H_2-10	11	2.02, dt (6.9, 6.3)	129.8, CH
9-OH	–	–			–	–
10	1.57, 1.49, m	37.3, CH_2	H-9		1.32, m	130.0, CH
11	1.33, m	25.4, CH_2			1.32, m	27.4, CH_2
12, 13	1.30, m	29.1, CH_2			1.32, m	29.4, CH_2
14	1.30, m	31.9, CH_2			1.32, m	31.9, CH_2
15	1.31, m	22.5, CH_2	H_3-16	14, 16	1.32, m	22.5, CH_2
16	0.9, t (7.0)	14.1, CH_3	H_2-15	14, 15	0.9, t (7.0)	14.0, CH_3

[a] ^{13}C values were deduced from HSQC and HMBC spectra.
[b] Multiplicity derived from the HSQC spectrum.

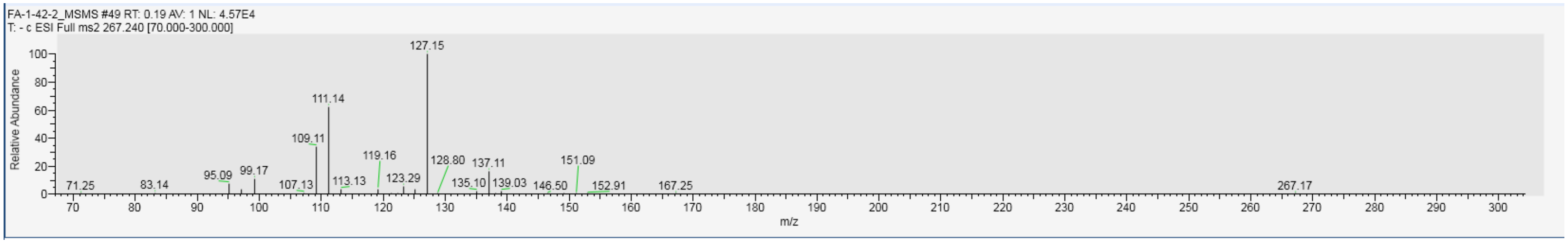

Figure S1. LRMS/MS spectra from the fragmentation of 7(*E*)-9-keto-hexadec-7-enoic acid (**1**) in the negative mode. The product ion scan parameters used were as follows: negative mode; scan rate (Da/sec):1000; used collision energy ramp (CER); Q1 and Q3 resolution (FWHM): 0.7; CID gas (mTorr): 1.5; chromatographic peak width: 6.

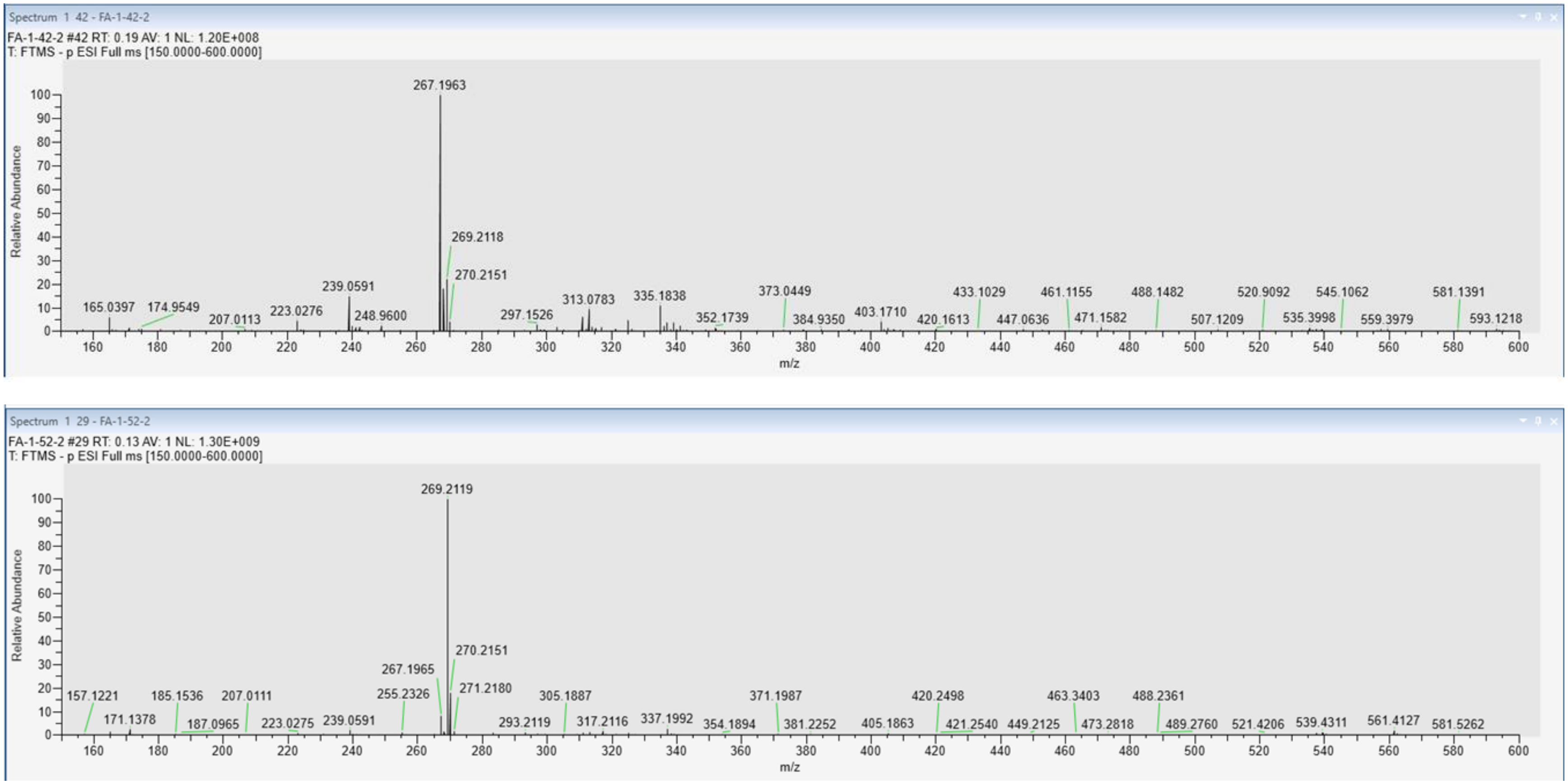

Figure S2. HRMS spectra of 7(*E*)-9-keto-hexadec-7-enoic acid (**1**) and (**2**) 7(*E*)-9-hydroxy-hexadec-7-enoic acid in the negative mode.

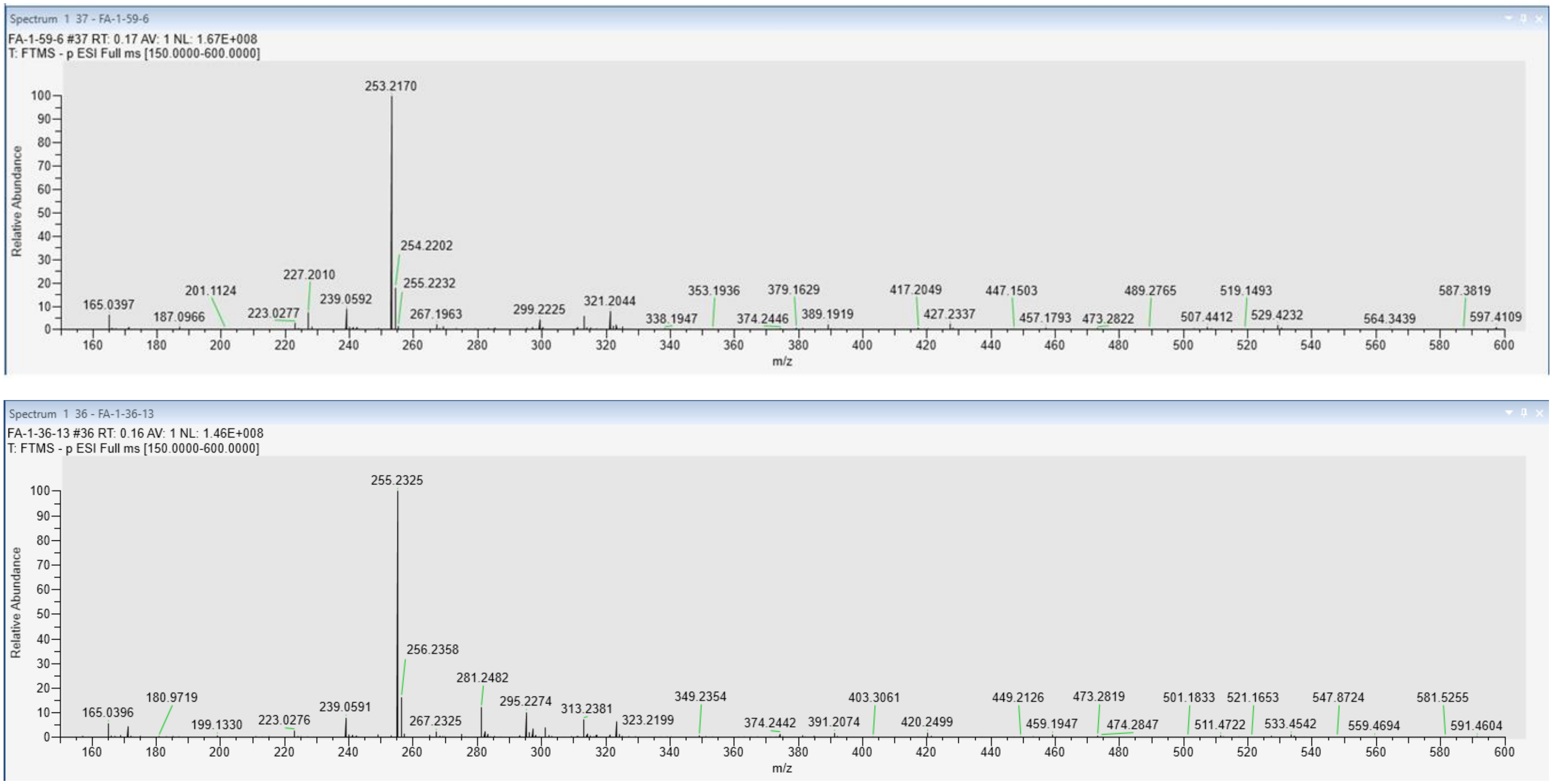

Figure S3. HRMS spectra of 9(*Z*)-hexadec-9-enoic acid (**3**) and hexadecanoic acid (**4**) in the negative mode

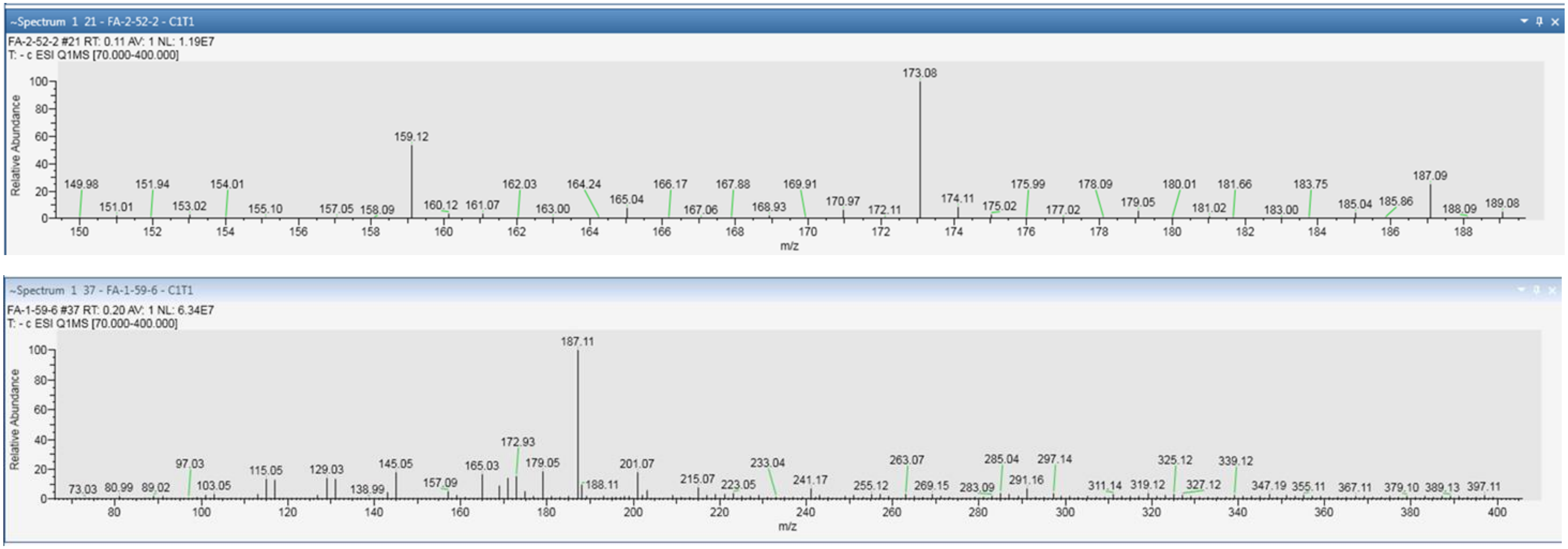

Figure S4. LRMS spectra of the ozonolysis products of compounds **2** and **3** in the negative mode.

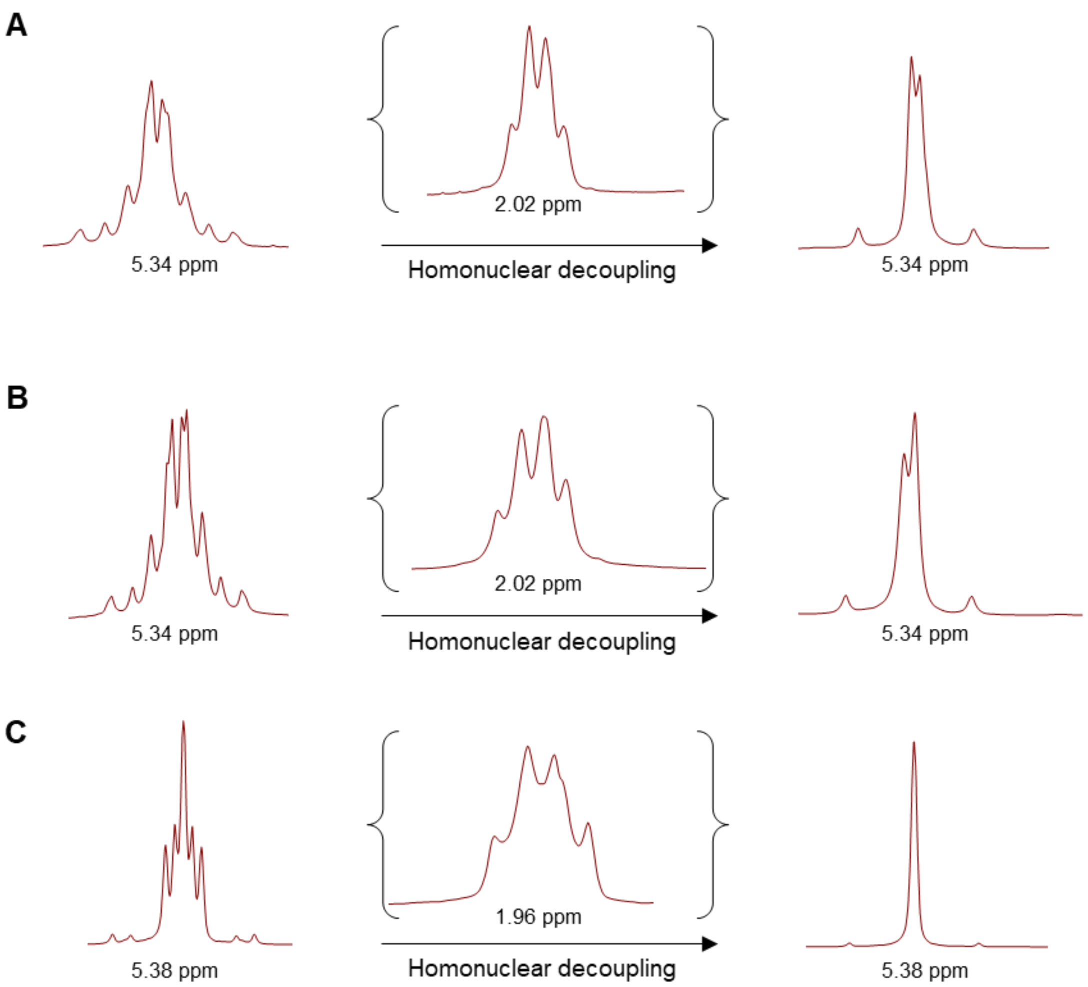

Figure S5. Homonuclear decoupling of allylic protons and the change in the ^{1}H NMR signals (600 MHz; $CDCl_3$) of olefinic protons before and after decoupling. A) 9(*Z*)-hexadec-9-enoic acid (**3**). B) Commercially available standard 9(*Z*)-hexadec-9-enoic acid. C) Commercially available standard 9(*E*)-hexadec-9-enoic acid. The allylic protons are shown between brackets.

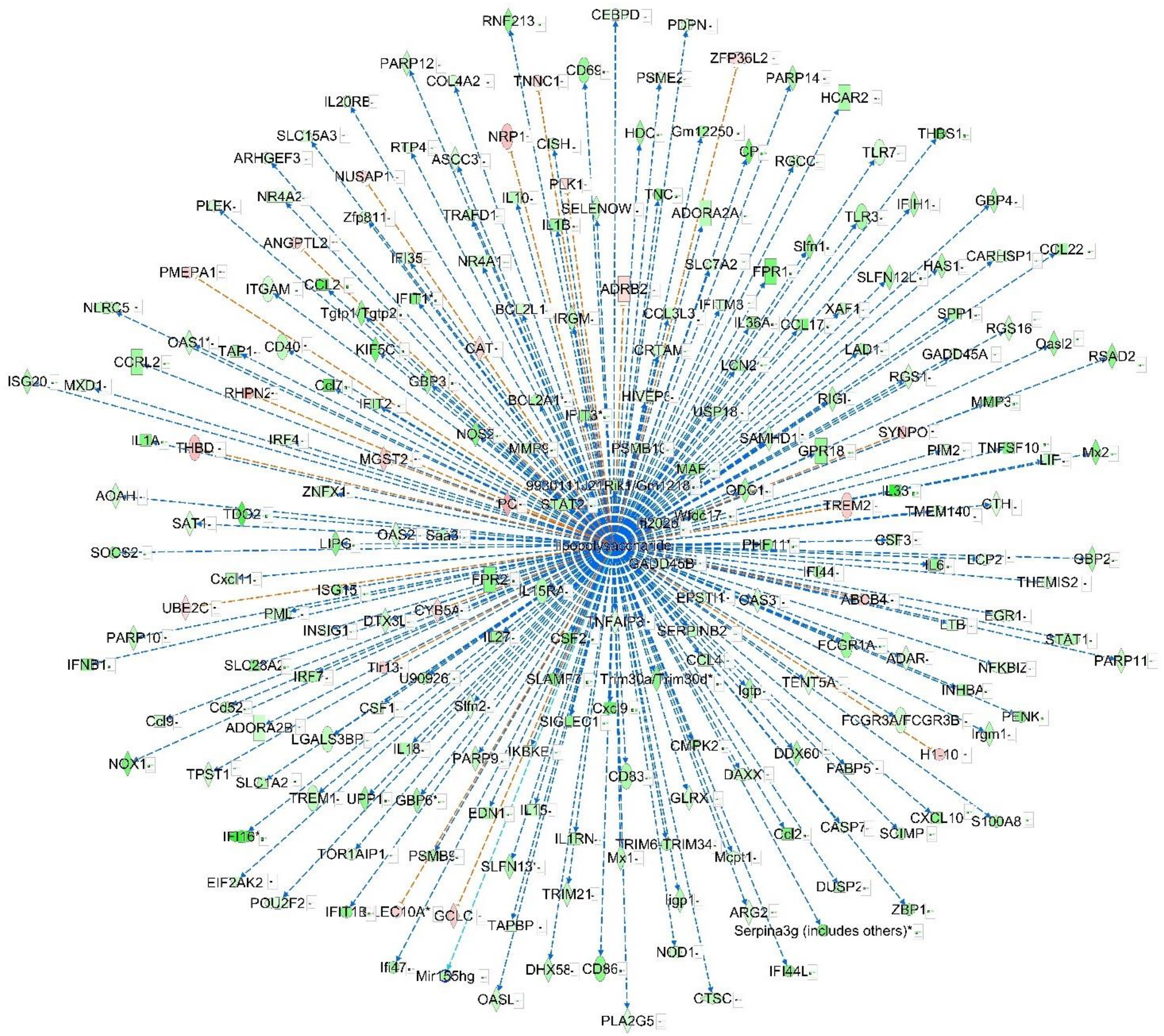

Figure S6. Upstream regulator network of LPS showing all genes with measurement direction consistent with its predicted inhibition state. Blue lines indicate inhibition while orange lines indicate activation.

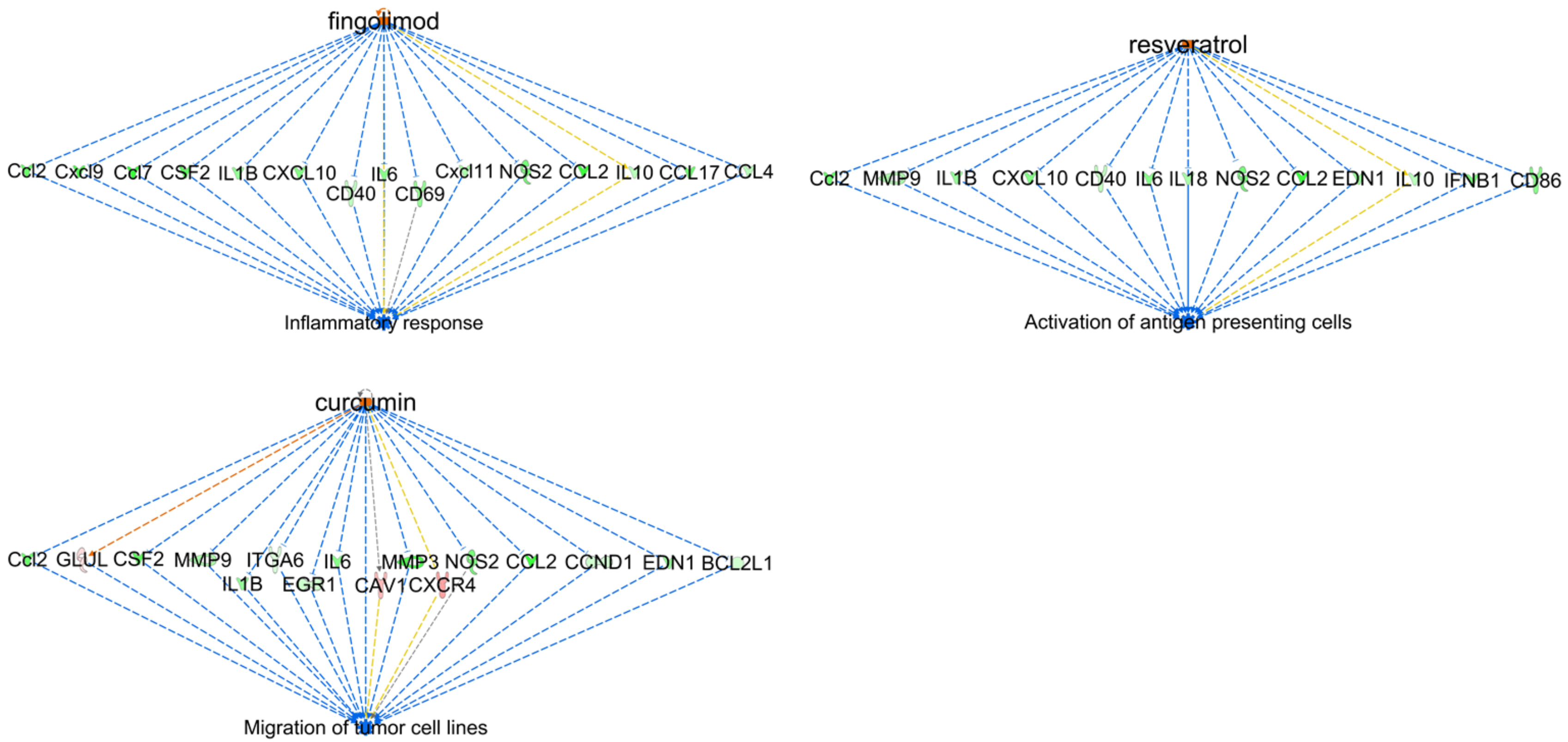

Figure S7. Regulator effect networks of the Nrf2 activators fingolimod, resveratrol, and curcumin predicted to be activated as it downregulates the same set of genes that are downregulated in the dataset in response to **1**. Blue lines indicate inhibition while orange lines indicate activation. Green nodes indicate downregulation while red nodes indicate upregulation.
